# Copper Overload Affects α‐Synuclein Clearance Mechanisms in a Parkinson's Disease In Vitro Model

**DOI:** 10.1002/adbi.202500274

**Published:** 2026-05-03

**Authors:** Debora Musarò, Marina Damato, Chiara Coppola, Marco Greco, Michele Maffia

**Affiliations:** ^1^ Laboratory of Clinical Proteomic “V Fazzi” Hospital Lecce Italy; ^2^ Laboratory of General and Human Physiology Department of Experimental Medicine University of Salento Lecce Italy

**Keywords:** alpha‐synuclein, autophagy, copper, Parkinson's disease

## Abstract

Parkinson's disease (PD) is a common neurodegenerative disorder characterized by the loss of dopaminergic neurons in the substantia nigra pars compacta and the formation of Lewy bodies, abnormal protein aggregates primarily composed of α‐synuclein. Copper, an essential trace element, plays a role in α‐synuclein aggregation and PD pathogenesis. This study examines the effects of copper overload on α‐synuclein clearance pathways, focusing on autophagy and the ubiquitin‐proteasome system (UPS) in dopaminergic SH‐SY5Y neuroblastoma cells. Copper exposure enhances autophagosome formation, as indicated by increased Beclin‐1 and LC3‐II levels, and impairs autophagic flux, evidenced by LC3‐II accumulation in the presence of chloroquine. Concurrently, copper increases polyubiquitinated proteins, suggesting UPS dysfunction, which is confirmed through MG132 treatment. These disruptions lead to the accumulation and aggregation of α‐synuclein, particularly in its phosphorylated form. Immunofluorescence reveals neurite‐localized α‐synuclein aggregates, consistent with copper's role in α‐synuclein pathology. This study highlights copper dyshomeostasis as a contributor to impaired α‐synuclein clearance through autophagy and UPS dysfunction, advancing the understanding of PD's molecular basis.

## Graphical abstract

This study reveals that copper overload disrupts neuronal protein clearance, leading to the accumulation of α‐synuclein and its phosphorylated form (p‐S129). By blocking autophagy and impairing the ubiquitin–proteasome system, copper promotes toxic aggregation in dopaminergic cells. These findings highlight copper dyshomeostasis as a key driver of Parkinson's disease progression through impaired proteostasis.

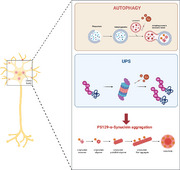

## 1 Introduction

Parkinson's disease (PD) is a neurodegenerative disorder that affects over 10 million people worldwide, primarily characterized by the progressive loss of dopaminergic neurons within the substantia nigra pars compacta (SNpc) [1]. The neuronal loss in the SNpc and the presence of intracellular inclusions, known as Lewy bodies (LBs), containing aggregates of α‐synuclein in the remaining dopaminergic neurons, are the neuropathological characteristics of PD [2, 3]. LBs contain various molecular components such as ubiquitin, heat shock proteins, oxidized/nitrated proteins, cytoskeletal proteins, as well as proteasomal and lysosomal elements [4].

Α‐synuclein is a neuronal protein predominantly localized in the presynaptic terminals of the striatum, hippocampus, thalamus, cerebellum, and neocortex [5]. Its function in a healthy brain includes regulation of the maintenance and recycling of synaptic vesicles, as well as a role in synaptic plasticity [6]. Inside LBs, α‐synuclein can adopt an amyloid‐like filamentous structure, become abnormally phosphorylated, and aggregate [7].

The protein can undergo numerous post‐translational modifications (PTMs); phosphorylation at serine 129 (p‐S129) has emerged as a hallmark of PD and related synucleinopathies [8].

However, accumulating evidence indicates that non‐phosphorylated species, including oligomeric, pre‐fibrillar, and even monomeric α‐synuclein, may also contribute to pathogenic mechanisms, suggesting that multiple conformations of the protein, rather than phosphorylation alone, drive neurodegeneration in PD [9, 10].

In addition to biosynthesis, folding, and PTMs, the cellular levels of α‐synuclein depend on its degradation and clearance via autophagy (dominant‐type lysosome) and the ubiquitin‐proteasome system (UPS) mechanisms [11]. The monomeric α‐synuclein is normally degraded by lysosomes through chaperone‐mediated autophagy (CMA) or UPS, whereas α‐synuclein aggregates displaying a longer half‐life are degraded by macroautophagy [12].

Both autophagy and proteasomal removal are necessary for maintaining α‐synuclein homeostasis in neurons, but the relative contribution of each pathway in PD remains incompletely established [13, 14, 15]. Despite extensive research, the mechanisms underlying α‐synuclein aggregation and its neurotoxic effects remain incompletely understood, posing a major challenge for developing disease‐modifying therapies. It has been proposed that a malfunction in the intracellular proteolytic mechanisms can lead to abnormal protein accumulation and aggregation [16, 17]. The maintenance of proteostasis is essential for neuronal survival and is tightly regulated by two primary protein degradation systems, autophagy and the UPS [18]. In PD, both systems are often disrupted, leading to the accumulation of misfolded and aggregated proteins, including α‐synuclein [19]. In addition to protein misfolding, mitochondrial dysfunction and resulting oxidative stress also contribute to neurodegeneration [20].

Copper is a trace element and is the third most abundant transition metal [21] required in several biological processes that include energy metabolism, antioxidant defense, iron metabolism, and the synthesis of neurotransmitters [22].

The imbalance of copper homeostasis has been identified as a factor leading to the development of idiopathic forms of PD [23]. In PD, the increase of free copper is associated with increased oxidative stress, α‐synuclein oligomerization, and aggregation in LBs [24]. Studies showed that rats exposed to copper (1 mg/L) presented not only liver damage, but also an increase of 50% in the brain metal content, diminished GSH, and lowered SOD activity [25]. In humans, long‐term exposure to copper in the workplace is also associated with an increased risk of developing PD [26]. The molecular link between copper dyshomeostasis and PD is not fully understood, but hypotheses have been suggested, which are based on some similarities of the clinical manifestations of PD with other copper‐related diseases, and on the ability of α‐synuclein to bind specifically metal [27, 28, 29, 30]. Some studies suggest that copper exacerbates proteostasis disruption, leading to α‐synuclein aggregation, whereas others propose that compensatory autophagy mitigates proteasomal deficits [12, 31, 32]. These conflicting findings highlight the need for further investigation into copper's impact on α‐synuclein clearance pathways.

In this study, we aimed to elucidate the effects of copper overload on α‐synuclein clearance mechanisms using SH‐SY5Y neuroblastoma cells differentiated into a dopaminergic‐like phenotype. This model system provides a valuable platform for studying the cellular processes underlying PD pathophysiology [33, 34]. By examining autophagy markers, UPS, and focusing in on p‐S129‐α‐synuclein accumulation, we provide evidence that copper disrupts proteostasis, impairing both clearance systems and contributing to α‐synuclein pathology. These findings underscore the toxic role of copper in PD and suggest that therapeutic strategies aimed at restoring metal homeostasis could hold promise for managing the disease.

## 2 Results

### 2.1 Cytotoxic Effect of Copper and Rotenone on Differentiated SH‐SY5Y

**FIGURE 1:**
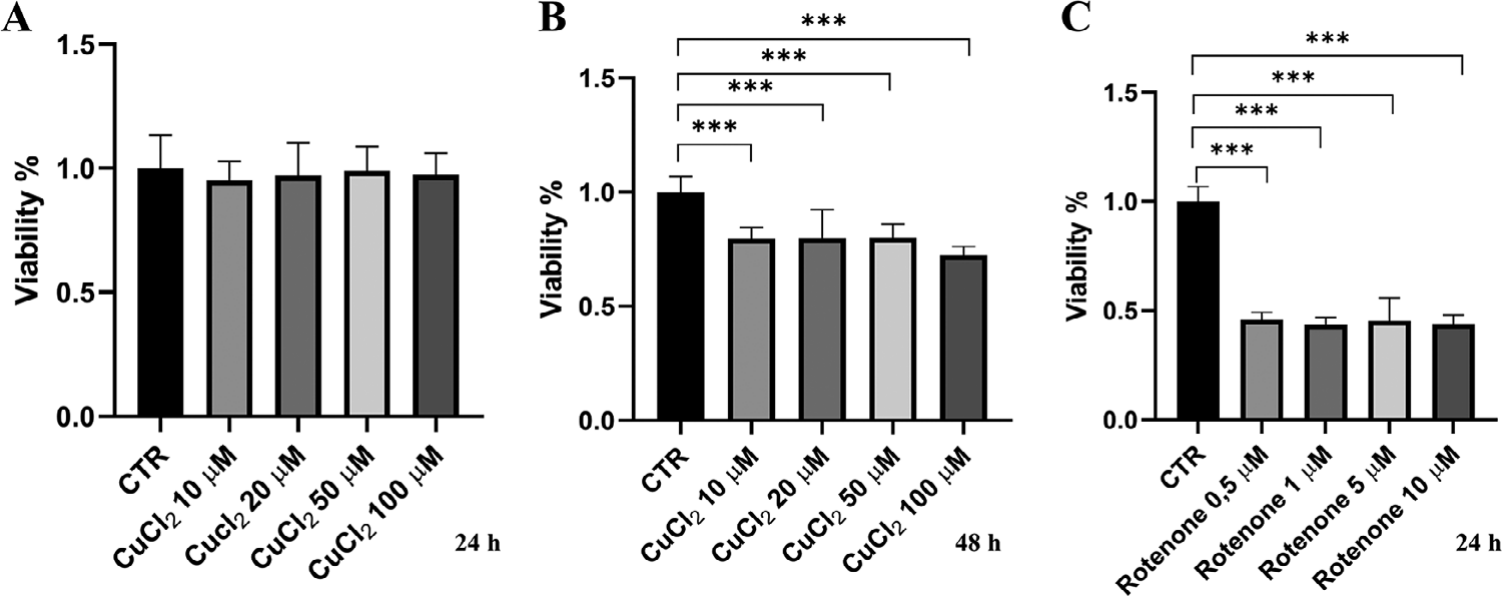
Effects of copper and rotenone on cell viability. Differentiated SH‐SY5Y cells were treated (A) with 10, 20, 50, or 100 µm CuCl_2_ for 24 h, (B) with the same concentrations of CuCl_2_ for 48 h, or (C) with 0.5, 1, 5, or 10 µm rotenone for 24 h. Data are presented as mean ± SD (*n* = 3). Statistical significance vs. untreated control (CTR) was assessed by one‐way ANOVA followed by Dunnett's post hoc test (α = 0.05). Analyses were performed using GraphPad Prism 8.0.1. ^***^ *p* < 0.0002.

Preliminary experiments were performed to determine the concentrations of copper chloride (CuCl_2_) and rotenone that induce cytotoxic effects in differentiated SH‐SY5Y cells. Rotenone, a well‐established oxidative stress inducer, is commonly used to create in vitro and in vivo models of PD [35]. Differentiated SH‐SY5Y cells were exposed to increasing concentrations of CuCl_2_ (10, 20, 50, and 100 µm) for 24 (Figure 1A) and 48 h (Figure 1B) and rotenone (0.5, 1, 5, and 10 µm) for 24 h (Figure 1C). While copper treatment did not impact cell viability after 24 h (Figure 1A), the MTT assay revealed a significant reduction in cell viability after 48 h of treatment, although the effect was not dose‐dependent (Figure 1B). In contrast, rotenone demonstrated a consistent toxic effect, reducing cell viability by approximately 60% across all tested concentrations after 24 h of treatment (Figure 1C). Based on these results, and to ensure consistency with previous studies [33], subsequent experiments were performed using 20 and 50 µm CuCl_2_ for 48 h. Rotenone treatments were carried out at 5 and 10 µm for 24 h, reflecting concentrations that produced a robust and reproducible cytotoxic effect.

### 2.2 Copper Modulates Autophagy in Differentiated SH‐SY5Y

**FIGURE 2:**
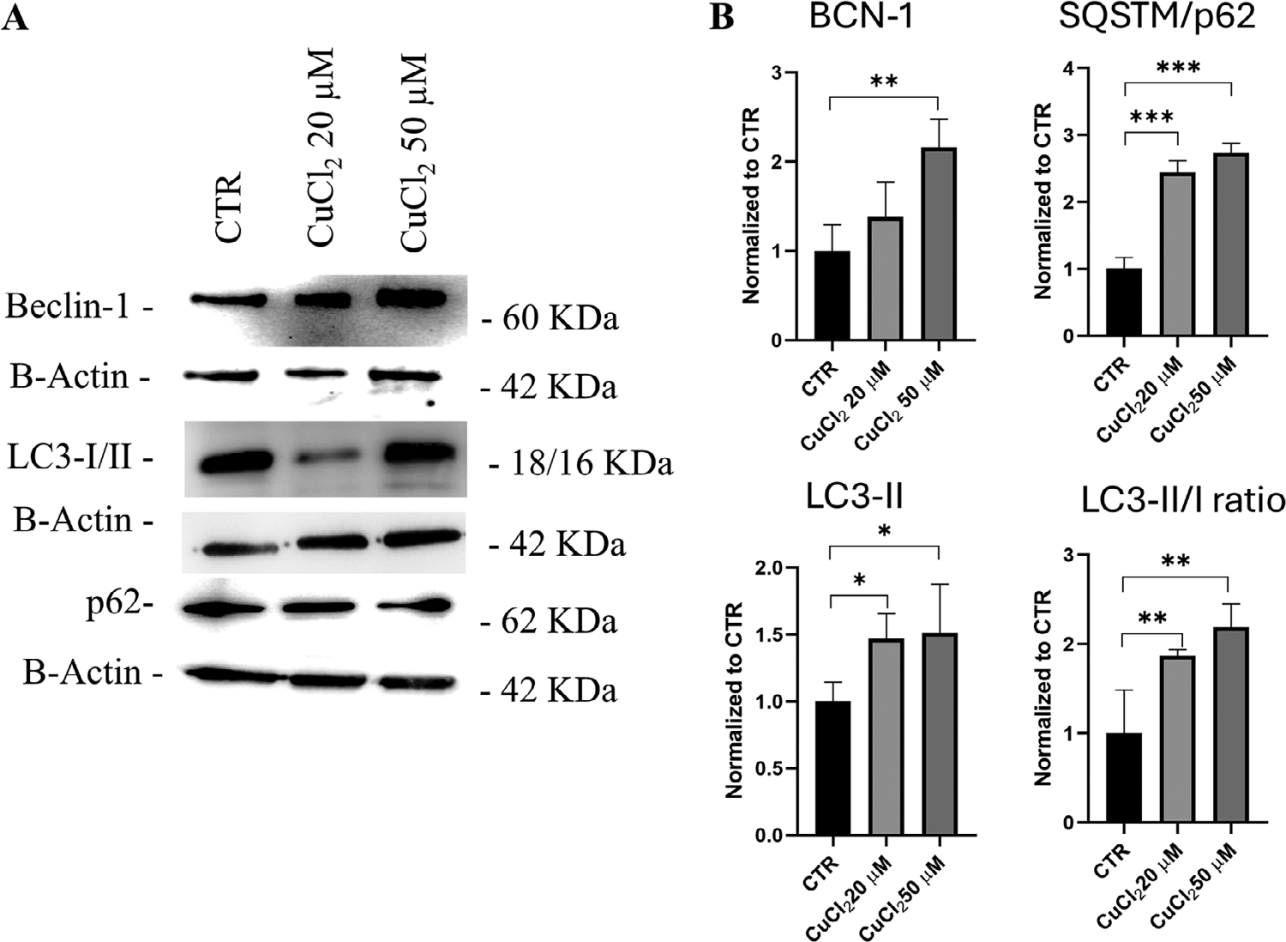
Effects of copper on autophagic markers. Differentiated SH‐SY5Y cells were treated with 20 or 50 µm CuCl_2_ for 48 h. (A) Representative immunoblot showing Beclin‐1, LC3‐I/II, and SQSTM1/p62 protein levels. (B) Densitometric quantification of Beclin‐1, LC3‐I/II, LC3‐II/I ratio and SQSTM1/p62 levels normalized to β‐actin (*n* = 4). Data are presented as mean ± SD. Statistical significance vs. untreated control (CTR) was determined by one‐way ANOVA followed by Dunnett's post hoc test (α = 0.05). Analyses were performed using GraphPad Prism 8.0.1. ^*^ *p* < 0.0332; ^**^ *p* < 0.0021; ^***^ *p* < 0.0002.

To investigate the impact of copper on autophagy, we assessed Beclin‐1, LC3‐I/II, and the autophagy cargo receptor SQSTM1/p62 protein levels. Differentiated SH‐SY5Y were treated with 20 and 50 µm of CuCl_2_ for 48 h. Beclin‐1 expression was elevated in copper‐treated cells compared to controls, indicating the initiation of autophagy (Figure 2A,B). However, copper treatment also resulted in a significant increase in LC3‐II levels (autophagosome marker) and SQSTM1/p62 expression (Figure 2A,B), suggesting impaired autophagic degradation. The concomitant increase in Beclin‐1, LC3‐II, and p62 indicates the blockage of autophagy flux [36, 37].

**FIGURE 3:**
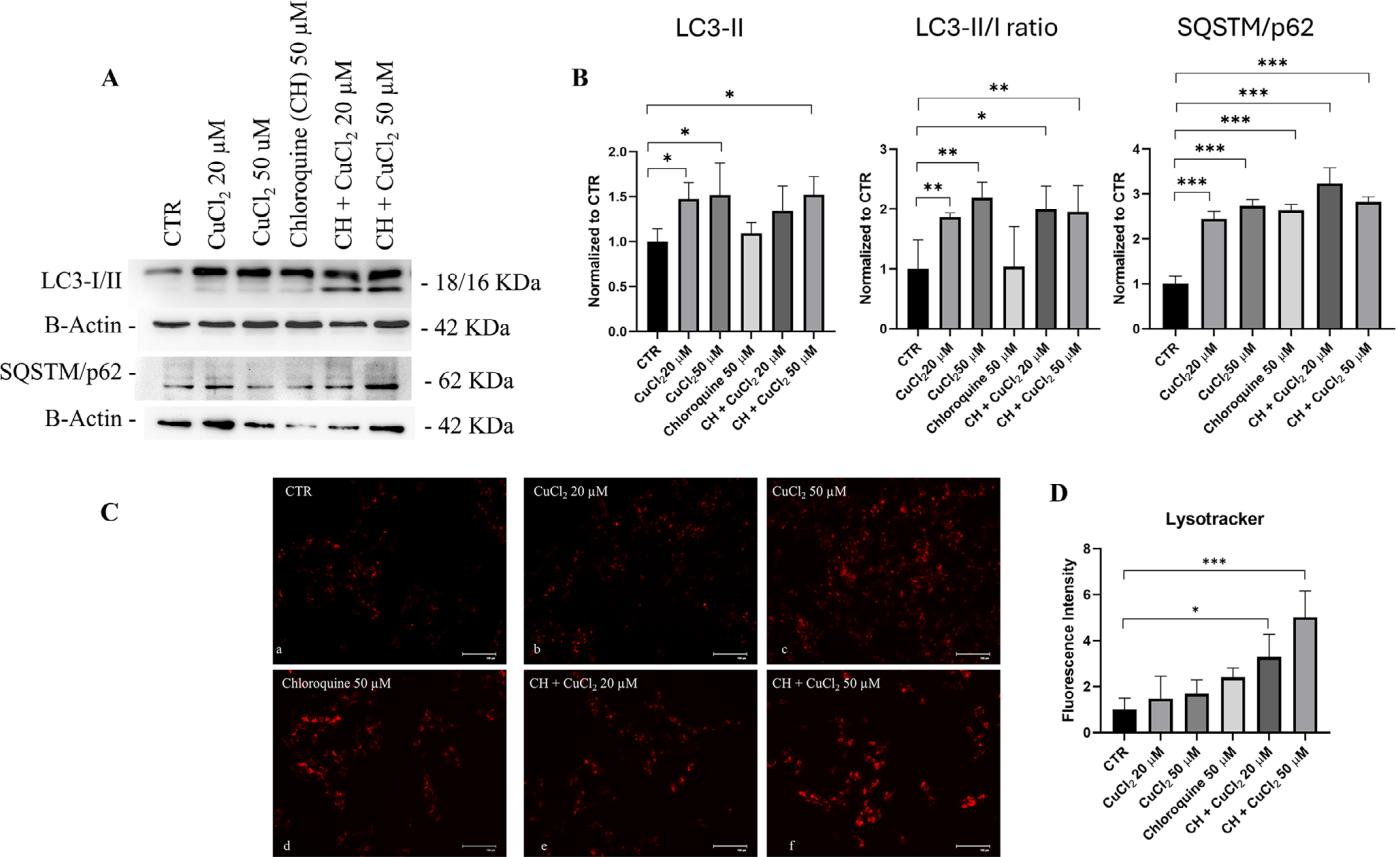
Copper induces autophagic flux blockage. Differentiated SH‐SY5Y cells were treated with 20 or 50 µm CuCl_2_ for 48 h in the absence or presence of chloroquine (CH, 50 µm, 1 h). (A) Representative immunoblot showing LC3‐I/II and SQSTM1/p62 protein levels. (B) Densitometric quantification of LC3‐I/II, LC3‐II/I ratio, and SQSTM1/p62 levels normalized to β‐actin (*n* = 4). Data are presented as mean ± SD. Statistical significance vs. untreated control (CTR) was determined by one‐way ANOVA followed by Dunnett's post hoc test (α = 0.05). Analyses were performed using GraphPad Prism 8.0.1. ^*^ *p* < 0.0332; ^**^ *p* < 0.0021; ^***^ *p* < 0.0002. (C) Representative fluorescence microscopy images (a–f) of cells stained with LysoTracker Red DND‐99 to mark lysosomes (red). Scale bar, 125 µm; magnification, 20×. (D) Graphical representation of the fluorescence intensity ratio LysoTracker Red DND‐99 normalized to CTR (*n* = 3).

To further examine autophagic flux, chloroquine (CH), an inhibitor of autophagosome‐lysosome fusion [38], was used at a concentration of 50 µm for 1 h before copper treatments. CH per se significantly increased LC3‐II and LC3‐II/I ratio. Cells treated with CH showed a further increase in LC3‐II, in LC3‐II/I ratio, and SQSTM/p62, with respect to CTR cells (Figure 3A,B). Additionally, we performed cell staining with lysotracker red to label lysosomes [39]. Cells treated for 48 h with 20 and 50 µm of CuCl_2_ showed increased red signal corresponding to lysosomes in comparison to CTR cells, consistent with impaired lysosomal function (Figure 3C **panel a v b and c**). Similar results were observed in CH‐treated cells, supporting the hypothesis that copper inhibits lysosome fusion with autophagosomes (Figure 3C **panel d v b and c**), supporting the hypothesis of impaired flux [38].

**FIGURE 4:**
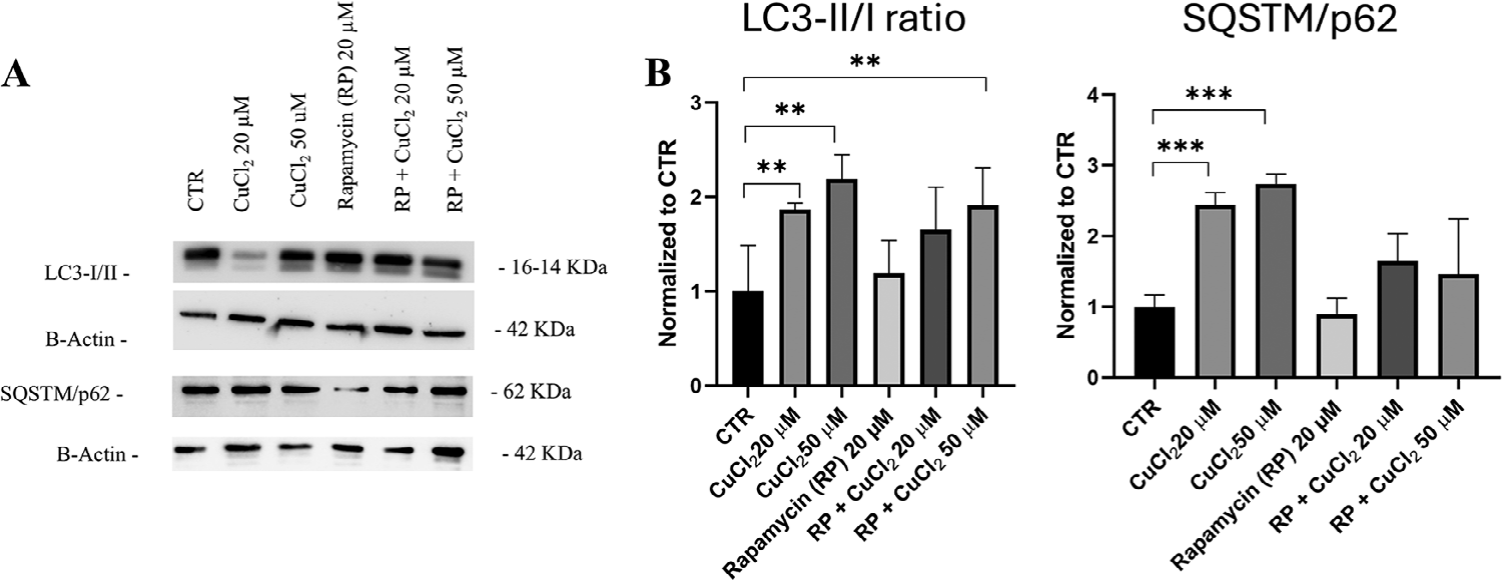
Rapamycin rescues autophagic flux. Differentiated SH‐SY5Y cells were treated with 20 or 50 µm CuCl_2_ for 48 h in the presence or absence of rapamycin (RP, 20 µm, 1 h). (A) Representative immunoblot showing LC3‐I/II and SQSTM1/p62 protein levels. (B) Densitometric quantification of the LC3‐II/I ratio and SQSTM1/p62 levels normalized to β‐actin (*n* = 4). Data are presented as mean ± SD. Statistical significance was assessed by one‐way ANOVA followed by Dunnett's post hoc test (α = 0.05). Analyses were performed using GraphPad Prism 8.0.1. ^*^ *p* < 0.0332; ^**^ *p* < 0.0021; ^***^ *p* < 0.0002 vs. untreated control (CTR).

In contrast, cells treated with 20 µm of rapamycin for 1 h showed an increase in the number of autophagosomes that were further increased after treatment with 20 and 50 µm of CuCl_2_. As expected, rapamycin alone increased the LC3‐II/I ratio and decreased SQSTM1/p62 protein levels. The combination of RP and copper produced the highest LC3‐II/I ratio, indicating a synergistic effect on autophagosome formation (Figure 4A,B). Overall, these results demonstrate that copper treatment leads to a block in autophagic flux in differentiated SH‐SY5Y cells.

### 2.3 Copper Impairs UPS

**FIGURE 5:**
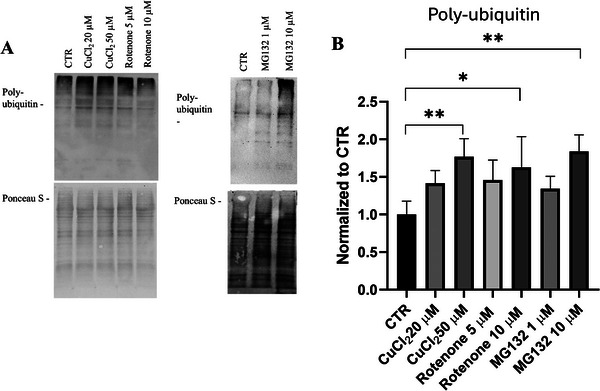
Polyubiquitin accumulation after CuCl_2_, rotenone, and MG132 treatments. Cells were treated with 20 or 50 µm CuCl_2_ for 48 h, or with 5 or 10 µm rotenone for 24 h. Cells were also treated with 1 or 10 µm MG132 for 1 h. (A) Representative immunoblot showing polyubiquitinated protein levels. (B) Densitometric quantification of polyubiquitinated proteins normalized to total protein (Ponceau S stain; *n* = 3). Data are mean ± SD. Statistical significance vs. untreated control (CTR) was assessed by one‐way ANOVA followed by Tukey's post‐hoc test (α = 0.05). Analyses were performed using GraphPad Prism 8.0.1. ^*^ *p* < 0.0332; ^**^ *p* < 0.0021.

To evaluate the functionality of the UPS, the accumulation of poly‐ubiquitinated proteins was measured following copper exposure. Differentiated cells were treated for 48 h with 20 and 50 µm of CuCl_2_. Western blot analysis showed a significant increase in poly‐ubiquitinated proteins in copper‐treated cells (Figure 5A,B), indicating proteasomal dysfunction. Similarly, treatment with rotenone at concentrations of 5 and 10 µm for 24 h resulted in elevated levels of poly‐ubiquitinated proteins, comparable to those observed with copper treatment (Figure 5A,B). Further confirmation of UPS impairment was provided by using MG132, a well‐established proteasome inhibitor [40, 41]. MG132 treatment produced effects similar to those of copper but only at a concentration of 10 µm, underscoring that copper impairs UPS.

### 2.4 Autophagy Modulates p‐S129‐α‐Synuclein Clearance in Copper‐Treated Cells

**FIGURE 6:**
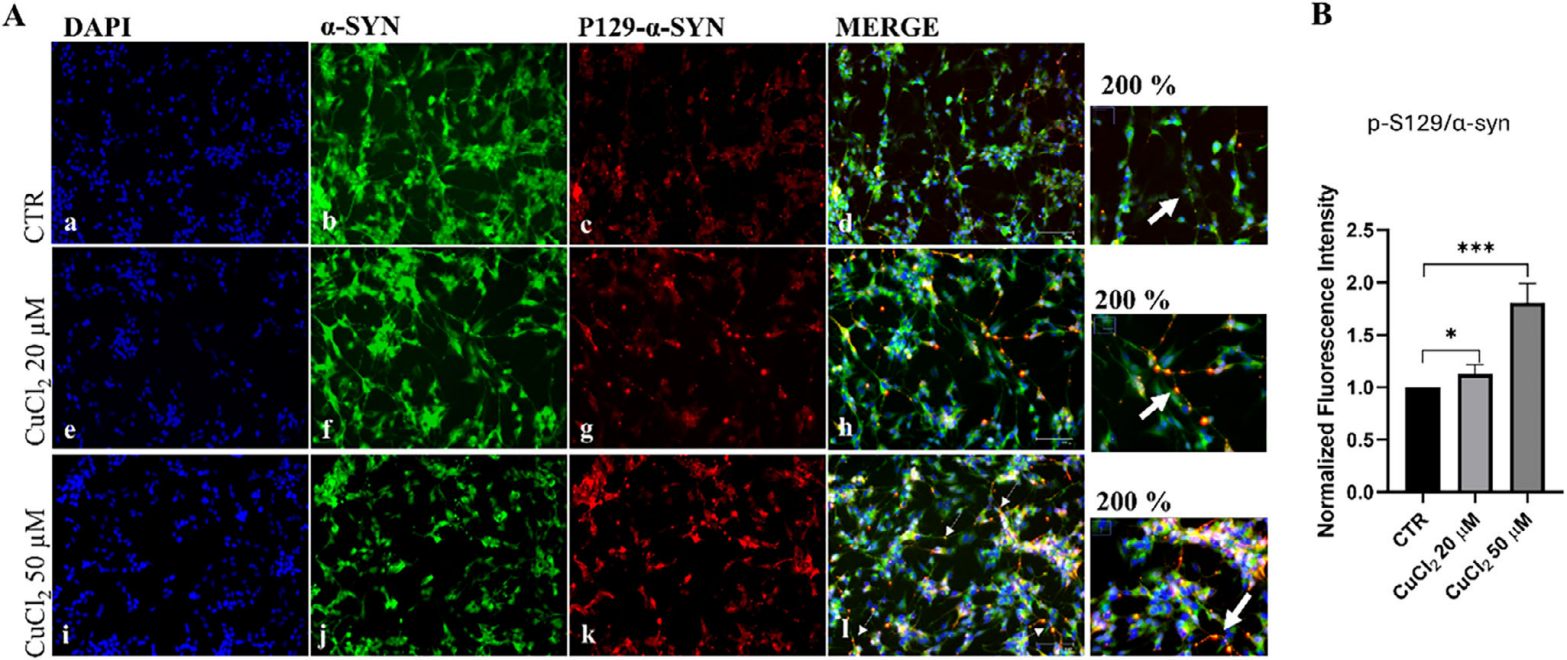
Effects of copper on α‐synuclein and phosphorylated α‐synuclein (p‐S129) localization. Cells were treated with 20 or 50 µm CuCl_2_ for 48 h. Representative fluorescence microscopy images (a–l) showing α‐synuclein (green) and p‐S129‐α‐synuclein (red). Merged images indicate colocalization (white arrows) of p‐S129‐α‐synuclein with α‐synuclein, with nuclei counterstained in blue. Magnification, 20×; scale bar, 125 µm. A selected region was enlarged 200% to better visualize colocalization. (B) Graphical representation of the fluorescence intensity ratio of p‐S129‐α‐synuclein to total α‐synuclein, normalized to nuclei (*n* = 3). Data are presented as mean ± SD. Statistical significance vs. untreated control (CTR) was assessed by one‐way ANOVA followed by Tukey's post‐hoc test (two‐sided, α = 0.05). Analyses were performed using GraphPad Prism 8.0.1. ^*^ *p* < 0.0332; ^***^ *p* < 0.0002.

Considering that the clearance of α‐synuclein and its phosphorylated form (p‐S129‐α‐synuclein) is linked to both autophagy and UPS [18, 42], its accumulation and cellular distribution were analyzed following treatments for 48 h of 20 and 50 µm of CuCl_2_, by immunofluorescence. In copper‐treated cells, α‐synuclein showed a diffuse cytoplasmatic distribution (Figure 6 panel b,f,j); on the contrary, p‐S129‐α‐synuclein expression was localized mainly in the neurite‐like cytoplasmic extensions (Figure 6 panel d,h,l).

**FIGURE 7:**
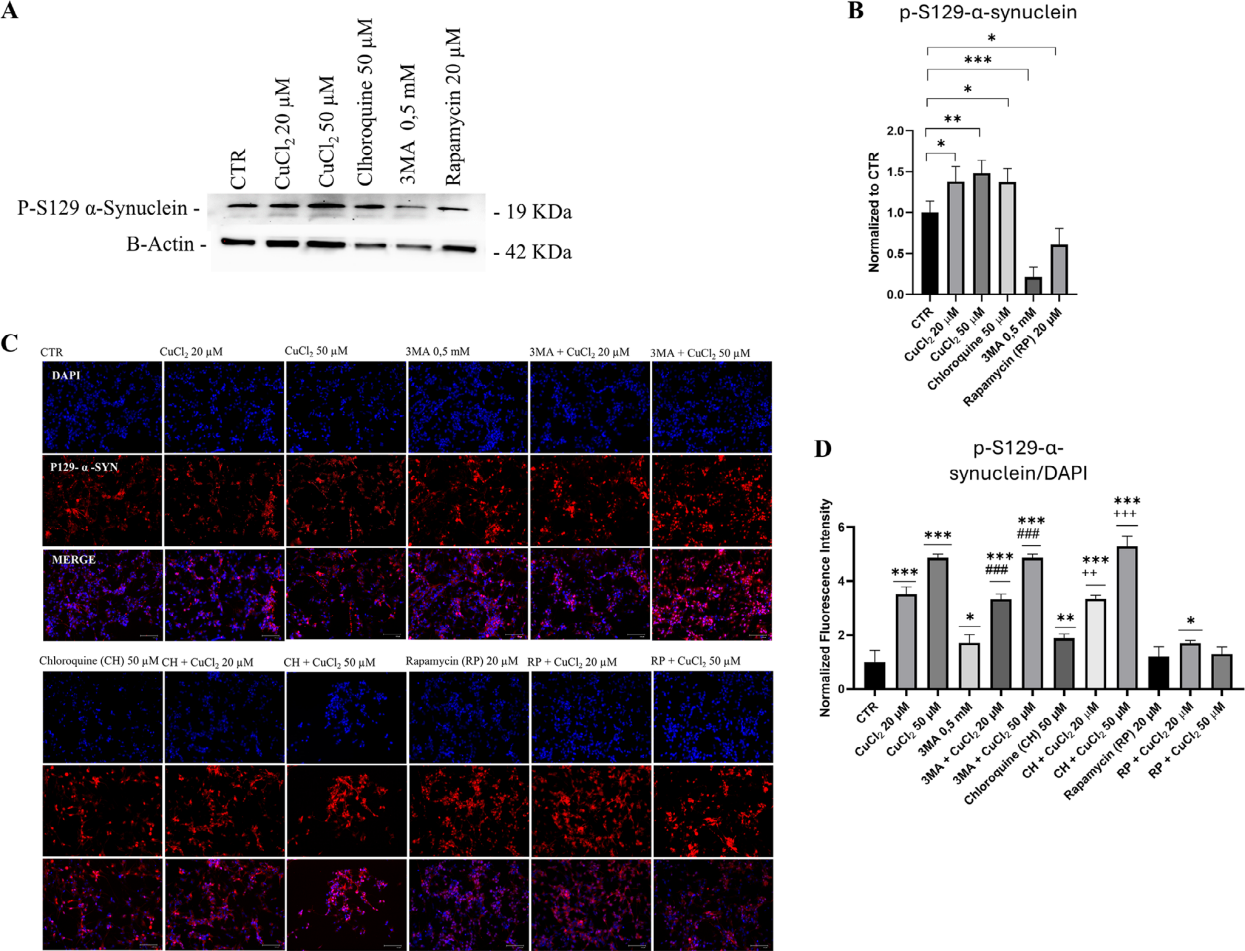
Effects of copper and autophagy modulators on p‐S129‐α‐synuclein. Differentiated SH‐SY5Y cells were treated with 20 or 50 µm CuCl_2_ for 48 h in combination with chloroquine (CH, 50 µm, 24 h), 3‐MA (0.5 mm, 24 h), or rapamycin (RP, 20 µm, 24 h). (A) Representative immunoblots showing p‐S129‐α‐synuclein protein levels. (B) Densitometric quantification of p‐S129‐α‐synuclein normalized to β‐actin (*n* = 4). (C) Representative fluorescence microscopy images of p‐S129‐α‐synuclein (red). Merged images show colocalization of p‐S129‐α‐synuclein with nuclei (blue). Magnification, 20×; scale bar, 125 µm. (D) Graphical representation of the fluorescence intensity of p‐S129‐α‐synuclein normalized to nuclei (*n* = 3). Data are presented as mean ± SD. Statistical significance vs. untreated control (CTR) was assessed by one‐way ANOVA followed by Tukey's post‐hoc test (α = 0.05). Analyses were performed using GraphPad Prism 8.0.1. ^*^ *p* < 0.0332; ^**^ *p* < 0.0021; ^***^ *p* < 0.0002. ^++^ *p*< 0.0021; ^+++^ *p*< 0.0002 vs. 3MA; ^###^ *p* < 0.0002 vs. CH.

We next aimed to determine whether modulating autophagy could reduce the accumulation of p‐S129‐α‐synuclein. Several studies have emphasized p‐S129 as a relevant pathological marker in synucleinopathies, given its strong association with α‐synuclein aggregation and LBs pathology [43, 44]. To test this, we treated cells with 50 µm chloroquine, 0,5 mm 3‐MA, or 20 µm rapamycin. After 24 h of treatment, p‐S129‐α‐synuclein protein levels remained significantly higher in chloroquine‐treated cells compared to untreated cells (Figure 7A,B). In contrast, neither 3‐MA nor rapamycin affected p‐S129‐α‐synuclein levels (Figure 7A,B).

Immunofluorescence staining for p‐S129‐α‐synuclein revealed that copper exposure significantly increased signal intensity compared to untreated control cells (Figure 7C,D). Treatment with both 20 and 50 µm CuCl_2_ resulted in a dose‐dependent accumulation of p‐S129‐α‐synuclein. Pharmacological inhibition of autophagy with 3‐MA or chloroquine further increased copper‐induced p‐S129‐α‐synuclein accumulation, with the combination of chloroquine and copper producing the most pronounced effect. Conversely, activation of autophagy with rapamycin significantly attenuated p‐S129‐α‐synuclein accumulation in copper‐treated cells, restoring the levels closer to those of untreated controls (Figure 7C,D). These results are in line with previous reports showing that autophagy activation promotes α‐synuclein clearance, whereas inhibition of autophagy enhances its accumulation [45, 46, 47].

## 3 Discussion

PD is the fastest‐growing neurological disorder, projected to double in incidence by 2040 [48]. Despite extensive research, available treatments only manage symptoms without halting disease progression. A central feature of PD pathology is α‐synuclein, a protein with diverse cellular roles, including vesicle recycling, dopamine metabolism, and metal binding [49]. α‐synuclein is an “intrinsically unfolded” protein localized in the cytosol, in presynaptic terminals near synaptic vesicles, or associated with the mitochondrial membrane [50]. Its specific role is not yet fully known; however, it has demonstrated the ability to interact with lipid membranes, recycle synaptic vesicles, metabolize dopamine, and also recognize with high affinity metals such as copper [51].

The binding between α‐synuclein and copper appears to be highly specific [52]. The presence of copper influences the stability of α‐synuclein dimers by inducing interchain bridges, stabilizing them, making each chain more extended and less flexible than metal‐free dimers, and potentially affecting aggregation [53]. Post‐translational modifications modulate this interaction: N‐terminal acetylation reduces copper (II) binding, while phosphorylation at Y125 and S129 enhances copper (II) affinity at the C‐terminal domain [42]. These modifications, along with oligomeric or prefibrillar α‐synuclein species, contribute to PD pathogenesis [10]. Given these molecular interactions, we investigated whether copper exposure affects proteostasis pathways in our cellular model.

Our data highlight copper's multifaceted impact on proteostasis, including autophagy and the UPS. The cytotoxicity assays confirmed that both copper chloride and rotenone significantly reduce cell viability, with rotenone exhibiting potent toxicity across all tested concentrations (Figure 1A–C). These findings are consistent with the established role of rotenone as a mitochondrial complex I inhibitor, known to impair oxidative phosphorylation and generate reactive oxygen species (ROS), leading to cell death [54]. To better understand the cellular mechanisms underlying this toxicity, we next examined how copper influences autophagic pathways.

Copper treatment affected autophagy, a conserved pathway essential for degrading misfolded proteins and damaged organelles [55]. Like other transition metals, excess copper perturbs autophagy regulation [56], suggesting a role in disrupting cellular homeostasis. Notably, systemic metabolic stress, such as that seen in type 2 diabetes, can similarly impair autophagic and mitophagic pathways, promoting protein aggregation and neurodegeneration [57]. Consistent with this, our experimental analyses revealed alterations in autophagy markers that point to defective flux.

During autophagy, when the phagophore is about to close, only LC3‐II is found in membrane‐bound form on the autophagosomal membrane, so the content of LC3‐II is directly proportional to the number of autophagosomes [58]. p62 often functions as a type of bridging protein in autophagy. This is because p62 can bind to LC3‐II, and the resulting complex is then degraded by lysosomes [59]. p62 accumulates upon autophagy inhibition, whereas it decreases when autophagy is induced. The observed upregulation of Beclin‐1, LC3‐II protein levels suggested enhanced autophagosome formation, indicative of autophagy initiation (Figure 2A,B). However, the concurrent increase in SQSTM7p62 protein, a substrate degraded by autophagy, implies a potential block in the late stages of autophagy, such as lysosomal fusion or degradation. This dysfunction was further corroborated by experiments with chloroquine, which exacerbated LC3‐II accumulation and LC3‐II/I ratio, indicating blocked lysosomal degradation (Figure 3A,B). LysoTracker Red DND‐99 staining confirmed the accumulation of acidic vesicles, suggesting a defect in lysosomal fusion, likely contributing to impaired autophagic flux (Figure 3C).

Lysosomes may attempt to compensate by increasing biogenesis, thereby sustaining degradative capacity. This process is regulated by TFEB, the master transcription factor for lysosomal and autophagy genes, which has also been implicated in PD pathogenesis [60].

Pharmacological modulation confirmed copper's disruptive effect on autophagy. Rapamycin, an mTOR inhibitor, increased the LC3‐II/I ratio and reduced p62, indicating strong autophagy induction (Figure 4A,B). However, co‐treatment with copper elevated p62 levels despite autophagy initiation, supporting the view that copper blocks autophagic completion and promotes substrate accumulation. To further clarify copper's impact on proteostasis, we next examined its effects on the ubiquitin–proteasome system.

Recent studies indicate that copper influences proteasome activity [61]. Most protein breakdown in mammalian cells is catalysed by the 26S proteasome, which selectively hydrolyses proteins attached with ubiquitin chains. Proteasomal degradation is essential for cell viability, and proteasome inhibitors can induce apoptosis [62]. Given that impaired autophagy alone could not fully explain the observed proteostasis defects, we next assessed whether copper also interferes with the UPS.

Cells exposed to copper and rotenone accumulated poly‐ubiquitinated proteins, consistent with proteasome inhibition (Figure 5A,B). While rotenone likely contributed through ROS generation [33]. MG132 experiments confirmed that copper directly impaired proteasome activity. This dual disruption of autophagy and UPS prompted us to assess how it impacts α‐synuclein homeostasis, a central hallmark of PD.

Consistent with our findings, copper has been shown to directly interact with proteasomal subunits, impairing their catalytic activity independently of oxidative stress [63]. Specifically, Cu(II) ions can induce conformational changes in the 20S proteasome that promote gate closure and reduced peptidase activity [61, 63], while copper complexes selectively inhibit the 26S proteasome and induce apoptosis in cancer cells [64]. Moreover, recent evidence highlights a bidirectional relationship between copper homeostasis and the UPS, since copper not only inhibits proteasome function but also modulates ubiquitin enzymes and ubiquitin itself [65].

It has been shown that proteasome inhibition activates autophagy involving the inhibition of PI3K/Akt/mTOR signaling pathway [66], which is notorious for the negative regulation of autophagy [67]. Moreover, it has been demonstrated that copper alone is able to downregulate the PI3K/Akt/mTOR pathway [68]. Dysregulation of the PI3K/Akt/mTOR pathway, such as suppressed mTOR, is commonly reported in brains and dopaminergic neurons from PD patients and contributes to the loss of dopaminergic neurons in PD [69].

Copper exposure increased α‐synuclein levels, particularly phosphorylated p‐S129 forms, normally degraded by the UPS in a ubiquitin‐independent manner [70]. Immunofluorescence revealed neurite‐localized puncta of p‐S129‐α‐synuclein (Figure 6A,B), consistent with metal‐induced aggregation [71].

Modulation of autophagy has differential effects on p‐S129‐α‐synuclein accumulation, depending on the specific pathway targeted. Unlike chloroquine, 3‐MA (an early‐stage autophagy inhibitor) and rapamycin did not alter p‐S129‐α‐synuclein levels compared to control cells (Figure 7A,B).

Immunofluorescence staining revealed that in the presence of copper, 3‐MA moderately enhanced p‐S129 levels, suggesting that blocking autophagy initiation reduces the cell's ability to clear phosphorylated α‐synuclein. Late‐stage inhibition with chloroquine produced a more pronounced effect, particularly in combination with copper, indicating that lysosomal dysfunction critically limits the degradation of p‐S129‐α‐synuclein species (Figure 7C,D). These findings support the notion that the completion of autophagic flux, rather than its initiation alone, is essential for maintaining α‐synuclein proteostasis under stress conditions such as metal overload. Conversely, autophagy activation via rapamycin effectively mitigated the copper‐induced accumulation of p‐S129‐α‐synuclein, restoring levels closer to untreated controls (Figure 7C,D). This protective effect suggests that enhancing autophagic flux can counteract the pathological consequences of copper exposure, likely by promoting lysosomal degradation of phosphorylated α‐synuclein.

In conclusion, copper exposure promotes the accumulation of p‐S129‐α‐synuclein in neuronal cells, with autophagic efficiency determining the extent of this pathogenic accumulation. Inhibition of autophagy exacerbates, while activation mitigates, copper‐induced α‐synuclein accumulation, highlighting the critical role of lysosomal degradation in maintaining proteostasis. These findings highlight the interplay between metal dyshomeostasis and proteostasis pathways, suggesting that strategies aimed at enhancing autophagic flux and restoring metal balance could help prevent α‐synuclein aggregation in PD [52, 53, 72, 73].

Similarly, iron overload has been shown to impair Parkin‐mediated proteasomal degradation, leading to α‐synuclein accumulation and mitochondrial dysfunction, further highlighting the role of metal dyshomeostasis in PD pathogenesis [74].

Taken together, our findings provide strong evidence that copper disrupts both autophagy and UPS. Future studies, using direct assays of autophagosome–lysosome fusion, lysosomal function, and proteasome activity, will be required to definitively establish the underlying mechanisms. In particular, to conclusively determine whether copper specifically impairs autophagosome–lysosome fusion, future studies employing tandem LC3 fluorescence reporters (e.g., mCherry–GFP–LC3) will be essential. Compounds such as clioquinol, a metal chelator, have shown promise in reducing neurotoxicity and protecting dopaminergic neurons [75]. However, given the delicate balance required in copper regulation, therapies must carefully avoid adverse effects associated with both deficiency and overload, as seen in Menkes and Wilson's diseases [28, 76]. Future research should explore therapeutic strategies to modulate copper levels and restore metal homeostasis [22, 77, 78]. Innovative drug delivery systems, such as solid lipid nanoparticles carrying neuroprotective agents, could enhance cellular uptake and target α‐synuclein accumulation, while modulating metal homeostasis and proteostatic pathways, offering a promising avenue for PD therapy [34].

To improve translational relevance, studies should employ physiologically relevant models such as human iPSCs or organoids [79]. Investigating copper dyshomeostasis, autophagy, and UPS in vivo will be essential to unravel pathway crosstalk and identify therapeutic targets to halt or reverse PD progression.

## 4 Conclusion

In conclusion, these results underscore the complex relationship between copper dyshomeostasis and cellular mechanisms responsible for protein degradation. The observed increase in p‐S129‐α‐synuclein levels due to copper exposure highlights the vulnerability of the UPS in managing protein clearance under metal stress. Furthermore, our findings suggest a potential feedback loop in which α‐synuclein accumulation exacerbates the burden on the autophagy system. This accumulation could create a vicious cycle, in which impaired α‐synuclein clearance leads to further aggregation and toxicity, overwhelming cellular mechanisms designed to prevent such outcomes. Understanding these interactions provides valuable insights into the pathological processes underlying neurodegenerative diseases such as PD, where α‐synuclein aggregation and impaired proteostasis are well‐established hallmarks of the disease.

## 5 Experimental Section/Methods

### 5.1 Cell Cultures and Treatments

The Human neuroblastoma SH‐SY5Y cell line was purchased from the European Collection of Cell Culture (ECCC, Salisbury, UK) and grown in high‐glucose Dulbecco's Modified Eagle Medium (DMEM) (Sigma, St. Louis, MO, USA) supplemented with 1 mm sodium pyruvate (Gibco, New York, NY, USA) and 10% (v/v) fetal bovine serum (FBS) (Sigma) in an incubator, at 37°C in a 5% CO_2_ atmosphere. Neuronal differentiation was induced as previously described [33, 80]. Briefly, cells were seeded at a density of 3 × 10^4^ cells/cm^2^ and cultured for 6 days in DMEM supplemented with 2% FBS and 10 µm all‐trans retinoic acid (RA; Sigma). The medium was then replaced with DMEM containing 1% FBS and 10 µm RA for an additional 3 days. In the final stage (5 days), cells were switched to Neurobasal medium (Gibco) supplemented with 50 ng/mL brain‐derived neurotrophic factor (BDNF; PeproTech, Rocky Hill, NJ, USA) and 1× B27 supplement (Gibco). Differentiated cultures displayed elongated neuritic processes and neuronal morphology, as expected.

All chemicals used in this study were dissolved in dimethyl sulfoxide (DMSO) except for CuCl_2_, Chloroquine, and 3‐methyladenine (3‐MA), which were suspended in water. The following chemical compounds were used for the treatment at the following concentration: CuCl_2_ (10, 20, 50, and 100 µm) (Sigma), Chloroquine (50 µm) (Sigma), 3‐MA (0,5 mm) (Selleckchem), Rapamycin (20 µm), Rotenone (0,5, 1, 5 and 10 µm) (Sigma), MG‐132 (1 and 10 µm).

### 5.2 Cell Viability

Cell viability was evaluated by 3‐(4,5‐dimethylthiazol‐2‐yl)‐5‐(3‐carboxymethylphenyl)‐2‐(4‐sulphophenyl)‐2H‐tetrazolium MTT assay (Sigma) used to assess metabolic activity [81]. Cells were seeded in a 96‐well plate (Becton, Dickinson and Company, Franklin Lakes, NJ, USA) and incubated overnight at 37°C in a 5% CO_2_ atmosphere. After treatments, the medium was substituted with a 0.5 mg/mL solution of MTT (Sigma) in fresh medium and incubated for 1 h at 37°C. Subsequently, the dark, purple‐colored formazan crystals formed in the viable population of cells were further solubilized in 100% DMSO. The dark purple solution obtained was quantified by measuring the absorbance at 595 nm utilizing a Multiskan FC Microplate Photometer (Thermo Fisher Scientific, Waltham, MA, USA). A subsequent measurement at 620 nm was undertaken for the purpose of background subtraction. Results were expressed as percentages of reduced MTT, assuming the absorbance of control cells as 100%.

### 5.3 Western Blotting

Immunoelectrophoresis was performed on cell extracts obtained by treating cells with ice‐cold RIPA buffer (Cell Signalling Technologies, Danvers, MA, USA) and centrifuged at 12 000 rpm for 20 min at 4°C. Protein extracts were quantified with Bradford's method, and approximately 25 µg of the lysate was loaded on SDS–PAGE gels. After the electrophoretic separation, proteins were transferred onto a PVDF membrane (Amersham Protean, GE Healthcare, Chalfont Buckinghamshire, UK). The membrane was blocked in Blotto A (Santa Cruz Biotechnology, Dallas, TX, USA) for 1 h, rinsed, and incubated with a primary antibody overnight at 4°C. After discarding the antibody, the membranes were washed with Tris‐buffered saline (TBS) and 0.05% Tween‐20 three times and then incubated with a proper HRP‐conjugated secondary antibody for 1 h at room temperature. After three further washes, protein–antibody complexes were visualized with the Clarity Western ECL Substrate (Bio‐Rad Laboratories, Hercules, CA, USA) and ChemiDoc Imaging System (Bio‐Rad Laboratories). Densitometric analyses were performed using the open‐source software ImageJ 1.48v (https://imagej.net/software/imagej, accessed on 28 September 2021) [82] to determine the optical density (OD) of the bands. The protein expression was normalized to β‐actin or to Ponceau S‐stain to account for variations in loading. The following primary antibodies were used (1:1000) and were purchased by Cell Signaling Technology: anti‐SQSTM1/p62, anti‐LC3A/B, Anti‐Beclin‐1(D40C5), Anti‐Phospho‐α‐Synuclein (Ser129) (D1R1R). Furthermore, the following primary antibodies were used: alpha Synuclein Antibody (211) (sc‐12767) (Santa Cruz Biotecnology), and Anti‐Ubiquitin (abcam). The following secondary antibodies were used (1:2000): anti‐rabbit IgG, HRP‐link antibody (Cell Signaling Technology) (Supporting Information).

### 5.4 Immunofluorescence

Cells were seeded and grown on coverslips adherent to the bottom of a 12‐well plate and incubated at 37°C. After the treatment, the cells were washed and fixed with 4% paraformaldehyde for 10 min at room temperature, then washed and blocked with blocking buffer (5% non‐fat dry milk in PBS) for 1 h at room temperature. The coverslips with cells were incubated with alpha Synuclein Antibody (211) (sc‐12767) primary antibody or with Anti‐Phospho‐α‐Synuclein (Ser129) (D1R1R) overnight at 4°C in gently shaking. Cells were then washed and incubated with Goat anti‐Mouse IgG (H+L) Cross‐Adsorbed Secondary Antibody, Alexa Fluor 488 (1:1000) (Thermo Fisher Scientific, Waltham, MA, USA) or Goat anti‐Rabbit IgG (H+L) Cross‐Adsorbed Secondary Antibody, Alexa Fluor 594 (1:1000) (Thermo Fisher Scientific, Waltham, MA, USA) for 1 h at room temperature; slides were protected from light, starting from this step to the end, by covering slides with aluminum foil. Then, they were washed in PBS three times and mounted using a mounting medium with DAPI (Fluoromount‐G Mounting Medium) (Thermo Fisher Scientific) and visualized with an EVOS Floid fluorescence microscope (Thermo Fisher Scientific). For Lysotracker stain, differentiated SH‐SY5Y cells were seeded at a density of 1 × 10^6^ cells/well in a 6‐well plate. After treatments, cells were incubated with LysoTracker red DND‐99 (50 nm) (Thermo Fisher Scientific) for 15 min, washed twice with PBS, and observed using the EVOS Floid fluorescence microscope (Thermo Fisher Scientific). Quantitative analysis of the immunofluorescence images was performed to calculate the average fluorescence intensity for measuring the gray values using ImageJ.

### 5.5 Statistical Analysis

The results were presented as mean ± standard deviation (SD) from at least three biological replicates. Multiple comparisons between groups were performed using one‐way analysis of variance (ANOVA). When ANOVA assumptions were met, either Dunnett's post hoc test or Tukey's post hoc test was applied. A significance threshold of *p* < 0.0332 was used. Statistical analyses were conducted using GraphPad Prism v8.0.1 (GraphPad Software Inc., La Jolla, CA, USA). The following statistical indicators were used throughout the text: ^*^ *p* < 0.0332^**^ *p* < 0.0021^***^ *p* < 0.0002.

## Conflicts of Interest

The authors declare no conflicts of interest.

## Supporting information




**Supporting file**: adbi70088‐sup‐0001‐SuppMat.pdf

## Data Availability

The data that support the findings of this study are available from the corresponding author upon reasonable request.

## References

[1] P. Prajjwal , H. S. Flores Sanga , K. Acharya , et al., “Parkinson's Disease Updates: Addressing the Pathophysiology, Risk Factors, Genetics, Diagnosis, along with the Medical and Surgical Treatment,” Annals of Medicine & Surgery 85, no. 10 (2023): 4887–4902. 37811009 10.1097/MS9.0000000000001142 PMC10553032

[2] D. W. Dickson , “Neuropathology of Parkinson Disease,” Parkinsonism & Related Disorders 46 (2018): S30–S33. 28780180 10.1016/j.parkreldis.2017.07.033 PMC5718208

[3] T. Koeglsperger , S. Rumpf , P. Schließer , et al., “Neuropathology of Incidental Lewy Body & Prodromal Parkinson's Disease,” Molecular Neurodegeneration 18, no. 1 (2023): 32, 10.1186/s13024-023-00622-7. 37173733 PMC10182593

[4] Q. Xia , L. Liao , D. Cheng , et al., “Proteomic Identification of Novel Proteins Associated with Lewy Bodies,” Frontiers in Bioscience 13, no. 10 (2008): 3850–3856. 18508479 10.2741/2973 PMC2663966

[5] J. Burré , M. Sharma , and T. C. Südhof , “Cell Biology and Pathophysiology of α‐Synuclein,” Cold Spring Harbor perspectives in medicine 8, no. 3 (2018): a024091. 28108534 10.1101/cshperspect.a024091 PMC5519445

[6] S. Bellani , V. L. Sousa , G. Ronzitti , F. Valtorta , J. Meldolesi , and E. Chieregatti , “The Regulation of Synaptic Function by α‐Synuclein,” Communicative & Integrative Biology 3, no. 2 (2010): 106–109, 10.4161/cib.3.2.10964. 20585500 PMC2889964

[7] M. Goedert , M. G. Spillantini , K. Del Tredici , and H. Braak , “100 years of Lewy Pathology,” Nature Reviews Neurology 9, no. 1 (2013): 13–24, 10.1038/nrneurol.2012.242. 23183883

[8] H. Fujiwara , M. Hasegawa , N. Dohmae , et al., “α‐Synuclein Is Phosphorylated in Synucleinopathy Lesions,” Nature Cell Biology 4, no. 2: 160–164, 10.1038/ncb748. 11813001

[9] C. Chavarría , S. Rodríguez‐Bottero , C. Quijano , P. Cassina , and J. M. Souza , “Impact of Monomeric, Oligomeric and Fibrillar Alpha‐Synuclein on Astrocyte Reactivity and Toxicity to Neurons,” Biochemical Journal 475, no. 19 (2018): 3153–3169. 30185433 10.1042/BCJ20180297

[10] B. A. Killinger , R. Melki , P. Brundin , and J. H. Kordower , “Endogenous Alpha‐Synuclein Monomers, Oligomers and Resulting Pathology: Let's Talk about the Lipids in the Room,” Npj Parkinson's Disease 5, no. 1 (2019): 23, 10.1038/s41531-019-0095-3. PMC6851126 31728405

[11] L. Stefanis , E. Emmanouilidou , M. Pantazopoulou , D. Kirik , K. Vekrellis , and G. K. Tofaris , “How Is Alpha‐Synuclein Cleared from the Cell?” Journal of Neurochemistry 150, no. 5 (2019): 577–590, 10.1111/jnc.14704. 31069800

[12] S. Sahoo , A. A. Padhy , V. Kumari , and P. Mishra , “Role of Ubiquitin–Proteasome and Autophagy‐Lysosome Pathways in α‐Synuclein Aggregate Clearance,” Molecular Neurobiology 59, no. 9 (2022): 5379–5407, 10.1007/s12035-022-02897-1. 35699874

[13] J. L. Webb , B. Ravikumar , J. Atkins , J. N. Skepper , and D. C. Rubinsztein , “α‐Synuclein Is Degraded by both Autophagy and the Proteasome,” Journal of Biological Chemistry 278, no. 27 (2003): 25009–25013. 12719433 10.1074/jbc.M300227200

[14] F. Limanaqi , F. Biagioni , S. Gambardella , P. Familiari , A. Frati , and F. Fornai , “Promiscuous Roles of Autophagy and Proteasome in Neurodegenerative Proteinopathies,” International Journal of Molecular Sciences 21, no. 8 (2020): 3028, 10.3390/ijms21083028. 32344772 PMC7215558

[15] D. Sepúlveda , M. Cisternas‐Olmedo , J. Arcos , M. Nassif , and R. L. Vidal , “Contribution of Autophagy‐Lysosomal Pathway in the Exosomal Secretion of Alpha‐Synuclein and Its Impact in the Progression of Parkinson's Disease,” Frontiers in Molecular Neuroscience 15 (2022): 805087. 35250476 10.3389/fnmol.2022.805087 PMC8891570

[16] D. Ebrahimi‐Fakhari , L. Wahlster , and P. J. McLean , “Protein Degradation Pathways in Parkinson's Disease: Curse or Blessing,” Acta Neuropathologica 124, no. 2 (2012): 153–172, 10.1007/s00401-012-1004-6. 22744791 PMC3417142

[17] Y. Li , S. Li , and H. Wu , “Ubiquitination‐Proteasome System (UPS) and Autophagy Two Main Protein Degradation Machineries in Response to Cell Stress ,” Cells 11, no. 5 (2022): 851. 35269473 10.3390/cells11050851 PMC8909305

[18] D. Ebrahimi‐Fakhari , I. Cantuti‐Castelvetri , Z. Fan , et al., “Distinct Roles in Vivo for the Ubiquitin‐Proteasome System and the Autophagy‐Lysosomal Pathway in the Degradation of α‐Synuclein,” Journal of Neuroscience 31, no. 41 (2011): 14508–14520. 21994367 10.1523/JNEUROSCI.1560-11.2011 PMC3587176

[19] T. Pan , S. Kondo , W. Le , and J. Jankovic , “The Role of Autophagy‐Lysosome Pathway in Neurodegeneration Associated with Parkinson's Disease,” Brain 131, no. 8 (2008): 1969–1978, 10.1093/brain/awm318. 18187492

[20] A. Y. Abramov , E. V. Potapova , V. V. Dremin , and A. V. Dunaev , “Interaction of Oxidative Stress and Misfolded Proteins in the Mechanism of Neurodegeneration,” Life 10, no. 7 (2020): 101, 10.3390/life10070101. 32629809 PMC7400128

[21] P. Szerdahelyi and P. Kása , “Histochemical Demonstration of Copper in Normal Rat Brain and Spinal Cord,” Histochemistry 85, no. 4 (1986): 341–347, 10.1007/BF00493487. 2428777

[22] M. Bisaglia and L. Bubacco , “Copper Ions and Parkinson's Disease: Why Is Homeostasis so Relevant?” Biomolecules 10, no. 2 (2020): 195, 10.3390/biom10020195. 32013126 PMC7072482

[23] M. N. Karpenko , Z. M. Muruzheva , E. Y. Ilyechova , P. S. Babich , and L. V. Puchkova , “Abnormalities in Copper Status Associated with an Elevated Risk of Parkinson's Phenotype Development,” Antioxidants 12, no. 9 (2023): 1654, 10.3390/antiox12091654. 37759957 PMC10525645

[24] S. Montes , S. Rivera‐Mancia , A. Diaz‐Ruiz , L. Tristan‐Lopez , and C. Rios , “Copper and Copper Proteins in Parkinson's Disease,” Oxidative Medicine and Cellular Longevity 2014 (2014): 1–15, 10.1155/2014/147251. PMC3941957 24672633

[25] D. Ozcelik and H. Uzun , “Copper Intoxication; Antioxidant Defenses and Oxidative Damage in Rat Brain,” Biological Trace Element Research 127, no. 1 (2009): 45–52, 10.1007/s12011-008-8219-3. 18784908

[26] S. Li , B. Ritz , Y. Gong , et al., “Proximity to Residential and Workplace Pesticides Application and the Risk of Progression of Parkinson's Diseases in Central California,” Science of the Total Environment 864 (2023): 160851. 36526213 10.1016/j.scitotenv.2022.160851 PMC11121507

[27] A. Piperno and M. Alessio , “Aceruloplasminemia: Waiting for an Efficient Therapy,” Frontiers in neuroscience 12 (2018): 903. 30568573 10.3389/fnins.2018.00903 PMC6290325

[28] A. Sánchez‐Monteagudo , E. Ripollés , M. Berenguer , and C. Espinós , “Wilson's Disease: Facing the Challenge of Diagnosing a Rare Disease,” Biomedicines 9, no. 9 (2021): 1100. 34572285 10.3390/biomedicines9091100 PMC8471362

[29] D. Atarod , F. Mamashli , A. Ghasemi , et al., “Bivalent Metal Ions Induce Formation of α‐Synuclein Fibril Polymorphs with Different Cytotoxicities,” Scientific Reports 12, no. 1 (2022): 11898, 10.1038/s41598-022-15472-4. 35831343 PMC9279330

[30] Y. Okita , A. N. Rcom‐H'cheo‐Gauthier , M. Goulding , R. S. Chung , P. Faller , and D. L. Pountney , “Metallothionein, Copper and Alpha‐Synuclein in Alpha‐Synucleinopathies,” Frontiers in neuroscience 11 (2017): 114. 28420950 10.3389/fnins.2017.00114 PMC5380005

[31] A. Anandhan , H. Rodriguez‐Rocha , I. Bohovych , et al., “Overexpression of Alpha‐Synuclein at Non‐Toxic Levels Increases Dopaminergic Cell Death Induced by Copper Exposure via Modulation of Protein Degradation Pathways,” Neurobiology of Disease 81 (2015): 76–92, 10.1016/j.nbd.2014.11.018. 25497688 PMC4459946

[32] M. Vidović and M. G. Rikalovic , “Alpha‐Synuclein Aggregation Pathway in Parkinson's Disease: Current Status and Novel Therapeutic Approaches,” Cells 11, no. 11 (2022): 1732. 35681426 10.3390/cells11111732 PMC9179656

[33] M. Greco , C. C. Spinelli , L. De Riccardis , et al., “Copper Dependent Modulation of α‐Synuclein Phosphorylation in Differentiated shsy5y Neuroblastoma Cells,” International Journal of Molecular Sciences 22, no. 4 (2021): 2038, 10.3390/ijms22042038. 33670800 PMC7922547

[34] R. Mallamaci , D. Musarò , M. Greco , et al., “Dopamine‐ and Grape‐Seed‐Extract‐Loaded Solid Lipid Nanoparticles: Interaction Studies between Particles and Differentiated SH‐SY5Y Neuronal Cell Model of Parkinson's Disease,” Molecules 29, no. 8 (2024): 1774, 10.3390/molecules29081774. 38675592 PMC11051794

[35] T. B. Sherer , R. Betarbet , A. K. Stout , S. Lund , M. Baptista , and A. V. Panov , “An in Vitro Model of Parkinson's Disease: Linking Mitochondrial Impairment to Altered α‐Synuclein Metabolism and Oxidative Damage,” Journal of Neuroscience 22, no. 16 (2002): 7006–7015. 12177198 10.1523/JNEUROSCI.22-16-07006.2002 PMC6757862

[36] J. M. Quiles , R. H. Najor , E. Gonzalez , et al., “Deciphering Functional Roles and Interplay between Beclin1 and Beclin2 in Autophagosome Formation and Mitophagy,” Science signaling 16, no. 770 (2023): abo4457. 10.1126/scisignal.abo4457 PMC10019900 36719945

[37] D. J. Klionsky , K. Abdelmohsen , A. Abe , et al., “Guidelines for the Use and Interpretation of Assays for Monitoring Autophagy,” Autophagy 12, no. 1 (2016): 1–222. 26799652 10.1080/15548627.2015.1100356 PMC4835977

[38] M. Mauthe , I. Orhon , C. Rocchi , et al., “Chloroquine Inhibits Autophagic Flux by Decreasing Autophagosome‐Lysosome Fusion,” Autophagy 14, no. 8 (2018): 1435–1455, 10.1080/15548627.2018.1474314. 29940786 PMC6103682

[39] D. C. Barral , L. Staiano , C. Guimas Almeida , et al., “Current Methods to Analyze Lysosome Morphology, Positioning, Motility and Function,” Traffic 23, no. 5 (2022): 238–269. 35343629 10.1111/tra.12839 PMC9323414

[40] H. K. Lee , S. H. Park , and M. J. Nam , “Proteasome Inhibitor MG132 Induces Apoptosis in human Osteosarcoma U2OS Cells,” Human & Experimental Toxicology 40, no. 11 (2021): 1985–1997, 10.1177/09603271211017972. 34002651

[41] W. Zhang , W. Xu , W. Chen , and Q. Zhou , “Interplay of Autophagy Inducer Rapamycin and Proteasome Inhibitor MG132 in Reduction of Foam Cell Formation and Inflammatory Cytokine Expression,” Cell Transplantation 27, no. 8 (2018): 1235–1248. 30001636 10.1177/0963689718786229 PMC6434468

[42] Y. Lu , M. Prudent , B. Fauvet , H. A. Lashuel , and H. H. Girault , “Phosphorylation of α‐Synuclein at Y125 and S129 Alters Its Metal Binding Properties: Implications for Understanding the Role of α‐Synuclein in the Pathogenesis of Parkinson's Disease and Related Disorders,” ACS Chemical Neuroscience 2, no. 11 (2011): 667–675, 10.1021/cn200074d. 22860160 PMC3369716

[43] J. Jiao , W. Liu , G. Gao , and H. Yang , “Serine‐129 Phosphorylated α‐Synuclein Drives Mitochondrial Dysfunction and Calcium Dysregulation in Parkinson's Disease Model,” Frontiers in Aging Neuroscience 17 (2025): 1538166, 10.3389/fnagi.2025.1538166. 40230488 PMC11994663

[44] N. Ramalingam and U. Dettmer , “α‐Synuclein serine129 Phosphorylation—The Physiology of Pathology,” Molecular Neurodegeneration 18, no. 1 (2023): 1–4, 10.1186/s13024-023-00680-x. 37953316 PMC10641962

[45] J. Jones‐Tabah , K. He , K. Senkevich , et al., “The Parkinson's Disease Risk Gene Cathepsin B Promotes Fibrillar Alpha‐Synuclein Clearance, Lysosomal Function and Glucocerebrosidase Activity in Dopaminergic Neurons,” BioRxiv (2023). 10.1186/s13024-024-00779-9 PMC11587650 39587654

[46] J. Gao , G. Perera , M. Bhadbhade , G. M. Halliday , and N. Dzamko , “Autophagy Activation Promotes Clearance of α‐Synuclein Inclusions in Fibril‐Seeded Human Neural Cells,” Journal of Biological Chemistry 294, no. 39 (2019): 14241–14256, 10.1074/jbc.RA119.008733. 31375560 PMC6768637

[47] Q. Xu , H. Wang , R. Yang , et al., “α‐Synuclein Amyloid Fibril Directly Binds to LC3B and Suppresses SQSTM1/p62‐Mediated Selective Autophagy,” Cell Research 35, no. 1 (2025): 72–75, 10.1038/s41422-024-01022-2. 39300253 PMC11701113

[48] E. R. Dorsey , T. Sherer , M. S. Okun , and B. R. Bloemd , “The Emerging Evidence of the Parkinson Pandemic,” Journal of Parkinsons Disease 8, no. s1 (2018): S3–S8. 10.3233/JPD-181474 PMC6311367 30584159

[49] A. T. Rodger , M. ALNasser , and W. G. Carter , “Are Therapies That Target α‐Synuclein Effective at Halting Parkinson's Disease Progression? A Systematic Review,” International Journal of Molecular Sciences 24, no. 13 (2023): 11022. 37446200 10.3390/ijms241311022 PMC10341763

[50] J. T. Bendor , T. P. Logan , and R. H. Edwards , “The Function of α‐Synuclein,” Neuron 79, no. 6 (2013): 1044–1066. 24050397 10.1016/j.neuron.2013.09.004 PMC3866954

[51] E. J. Byrd , M. Wilkinson , S. E. Radford , and F. Sobott , “Taking Charge: Metal Ions Accelerate Amyloid Aggregation in Sequence Variants of α‐Synuclein,” Journal of the American Society for Mass Spectrometry 34, no. 3 (2023): 493–504, 10.1021/jasms.2c00379. 36794792 PMC9983014

[52] X. Wang , D. Moualla , J. A. Wright , and D. R. Brown , “Copper Binding Regulates Intracellular Alpha‐Synuclein Localisation, Aggregation and Toxicity,” Journal of Neurochemistry 113, no. 3 (2010): 704–714, 10.1111/j.1471-4159.2010.06638.x. 20141569

[53] L. Savva and J. A. Platts , “Computational Investigation of Copper‐Mediated Conformational Changes in α‐Synuclein Dimer,” Physical Chemistry Chemical Physics 26, no. 4 (2024): 2926–2935, 10.1039/D3CP04697D. 38193190

[54] C. M. Tanner , F. Kame , G. W. Ross , et al., “Rotenone, Paraquat, and Parkinson's Disease,” Environmental Health Perspectives 119, no. 6 (2011): 866–872. 21269927 10.1289/ehp.1002839 PMC3114824

[55] A. Monaco and A. Fraldi , “Protein Aggregation and Dysfunction of Autophagy‐Lysosomal Pathway: a Vicious Cycle in Lysosomal Storage Diseases,” Frontiers in Molecular Neuroscience 13 (2020): 37. 32218723 10.3389/fnmol.2020.00037 PMC7079699

[56] S. Sahni , D. H. Bae , P. J. Jansson , and D. R. Richardson , “The Mechanistic Role of Chemically Diverse Metal Ions in the Induction of Autophagy,” Pharmacological Research 119 (2017): 118–127. 28087444 10.1016/j.phrs.2017.01.009

[57] M. Greco , A. Munir , D. Musarò , C. Coppola , and M. Maffia , “Restoring Autophagic Function: a Case for Type 2 Diabetes Mellitus Drug Repurposing in Parkinson's Disease,” Frontiers in Neuroscience 17 (2023): 1244022. 38027497 10.3389/fnins.2023.1244022 PMC10654753

[58] D. J. Klionsky , A. Kamal Abdel‐Aziz , S. Abdelfatah , et al., “Guidelines for the Use and Interpretation of Assays for Monitoring Autophagy (4th edition),” Autophagy 17: 1–382. 33634751 10.1080/15548627.2020.1797280 PMC7996087

[59] G. Bjørkøy , T. Lamark , S. Pankiv , A. Øvervatn , A. Brech , and T. Johansen , “Chapter 12 Monitoring Autophagic Degradation of p62/SQSTM1,” Methods in Enzymology 452 (2009): 181–197. 19200883 10.1016/S0076-6879(08)03612-4

[60] M. Decressac , B. Mattsson , P. Weikop , M. Lundblad , J. Jakobsson , and A. Björklund , “TFEB‐Mediated Autophagy Rescues Midbrain Dopamine Neurons from α‐Synuclein Toxicity,” Proceedings of the National Academy of Sciences 110, no. 19 (2013): E1817–E1826, 10.1073/pnas.1305623110. PMC3651458 23610405

[61] A. M. Santoro , I. Monaco , F. Attanasio , et al., “Copper(II) Ions Affect the Gating Dynamics of the 20S Proteasome: a Molecular and in Cell Study,” Scientific Reports 6 (2016): 33444. 27633879 10.1038/srep33444 PMC5025780

[62] E. E. Manasanch and R. Z. Orlowski , “Proteasome Inhibitors in Cancer Therapy,” Nature Reviews Clinical Oncology 14, no. 7 (2017): 417–433, 10.1038/nrclinonc.2016.206. PMC5828026 28117417

[63] K. G. Daniel , P. Gupta , R. H. Harbach , W. C. Guida , and Q. P. Dou , “Organic Copper Complexes as a New Class of Proteasome Inhibitors and Apoptosis Inducers in human Cancer Cells,” Biochemical Pharmacology 67, no. 6 (2004): 1139–1151. 15006550 10.1016/j.bcp.2003.10.031

[64] Z. Zhang , H. Wang , M. Yan , H. Wang , and C. Zhang , “Novel Copper Complexes as Potential Proteasome Inhibitors for Cancer Treatment,” Molecular Medicine Reports 15, no. 1 (2017): 3–11, 10.3892/mmr.2016.6022. 27959411

[65] B. Zhang and R. Burke , “Copper Homeostasis and the Ubiquitin Proteasome System,” Metallomics 15, no. 3 (2023): mfad010, 10.1093/mtomcs/mfad010. 36822629 PMC10022722

[66] B. Tang , J. Cai , L. Sun , et al., “Proteasome Inhibitors Activate Autophagy Involving Inhibition of PI3K‐Akt‐mTOR Pathway as an Anti‐Oxidation Defense in human RPE Cells,” PLoS ONE 9, no. 7 (2014): 103364. 10.1371/journal.pone.0103364 PMC4111584 25062253

[67] S. Giacoppo , P. Bramanti , and E. Mazzon , “Triggering of Inflammasome by Impaired Autophagy in Response to Acute Experimental Parkinson's Disease: Involvement of the PI3K/Akt/mTOR Pathway,” Neuroreport 28, no. 15 (2017): 996–1007. 28902711 10.1097/WNR.0000000000000871 PMC5610561

[68] L. J. Bain , L. Lu , Y. Zhang , et al., “The Role of Autophagy in Copper‐Induced Apoptosis and Developmental Neurotoxicity in SH‐SY5Y Cells,” Toxic 13 (2025): 63. 10.3390/toxics13010063 PMC11769067 39853061

[69] Y. Zhang , H. Guo , X. Guo , et al., “Involvement of Akt/mTOR in the Neurotoxicity of Rotenone‐Induced Parkinson's Disease Models,” International Journal of Environmental Research and Public Health 16, no. 20 (2019): 3811. 31658620 10.3390/ijerph16203811 PMC6843606

[70] Y. Machiya , S. Hara , S. Arawaka , et al., “Phosphorylated α‐Synuclein at Ser‐129 Is Targeted to the Proteasome Pathway in a Ubiquitin‐Independent Manner,” Journal of Biological Chemistry 285, no. 52 (2010): 40732–40744, 10.1074/jbc.M110.141952. 20959456 PMC3003373

[71] C. Bacchella , F. Camponeschi , P. Kolkowska , et al., “Copper Binding and Redox Activity of α‐Synuclein in Membrane‐Like Environment,” Biomolecules 13, no. 2 (2023): 287, 10.3390/biom13020287. 36830656 PMC9953312

[72] R. Wang , Y. Wang , L. Qu , et al., “Iron‐Induced Oxidative Stress Contributes to α‐Synuclein Phosphorylation and Up‐Regulation via Polo‐Like Kinase 2 and Casein Kinase 2,” Neurochemistry International 125 (2019): 127–135, 10.1016/j.neuint.2019.02.016. 30797969

[73] J. A. Castillo‐Gonzalez , M. D. J. Loera‐Arias , O. Saucedo‐Cardenas , R. Montes‐De‐Oca‐Luna , A. Garcia‐Garcia , and H. Rodriguez‐Rocha , “Phosphorylated α‐Synuclein‐Copper Complex Formation in the Pathogenesis of Parkinson's Disease,” Parkinsons Disease 2017 (2017): 9164754. 10.1155/2017/9164754 PMC5733240 29333317

[74] U. Ganguly , A. Banerjee , S. S. Chakrabarti , et al., “Interaction of α‐Synuclein and Parkin in Iron Toxicity on SH‐SY5Y Cells: Implications in the Pathogenesis of Parkinson's Disease,” Biochemical Journal 477, no. 6 (2020): 1109–1122. 32108853 10.1042/BCJ20190676

[75] M. Teil , E. Doudnikoff , M. L. Thiolat , S. Bohic , E. Bezard , and B. Dehay , “The Zinc Ionophore Clioquinol Reduces Parkinson's Disease Patient‐Derived Brain Extracts‐Induced Neurodegeneration,” Molecular Neurobiology 59, no. 10 (2022): 6245–6259. 35915387 10.1007/s12035-022-02974-5

[76] Z. Tümer and L. B. Møller , “Menkes Disease,” European Journal of Human Genetics 18, no. 5 (2010): 511–518. 19888294 10.1038/ejhg.2009.187 PMC2987322

[77] L. Chen , J. Min , and F. Wang , “Copper Homeostasis and Cuproptosis in Health and Disease,” Signal Transduction and Targeted Therapy 7, no. 1 (2022): 378. 36414625 10.1038/s41392-022-01229-y PMC9681860

[78] Y. An , S. Li , X. Huang , X. Chen , H. Shan , and M. Zhang , “The Role of Copper Homeostasis in Brain Disease,” International Journal of Molecular Sciences 23, no. 22 (2022): 13850, 10.3390/ijms232213850. 36430330 PMC9698384

[79] W. Zheng , Q. Li , C. Zhao , Y. Da , H. L. Zhang , and Z. Chen , “Differentiation of Glial Cells from hiPSCs: Potential Applications in Neurological Diseases and Cell Replacement Therapy,” Frontiers in Cellular Neuroscience 12 (2018): 239. 30140204 10.3389/fncel.2018.00239 PMC6094089

[80] M. M. Shipley , C. A. Mangold , and M. L. Szpara , “Differentiation of the SH‐SY5Y human Neuroblastoma Cell Line,” Journal of Visualized Experiments 2016, no. 108 (2016): 53193, 10.3791/53193. PMC4828168 26967710

[81] Z. Nozhat , M. S. Khalaji , M. Hedayati , and S. K. Kia , “Different Methods for Cell Viability and Proliferation Assay: Essential Tools in Pharmaceutical Studies,” Anti‐Cancer Agents in Medicinal Chemistry 22, no. 4 (2021): 703–712, 10.2174/1871520621999201230202614. 33390140

[82] C. A. Schneider , W. S. Rasband , and K. W. Eliceiri , “NIH Image to ImageJ: 25 Years of Image Analysis,” Nature Methods 9, no. 7 (2012): 671–675, 10.1038/nmeth.2089. 22930834 PMC5554542

