# Supplementary material for: Copper Overload Affects α‐Synuclein Clearance Mechanisms in a Parkinson's Disease In Vitro Model
Source: Adv Biol (Weinh). 2026 May 3;10:e00274. doi: 10.1002/adbi.202500274 (PMC13136777; doi:10.1002/adbi.202500274)
Supplement: Supplementary file 1 — Supporting file: adbi70088‐sup‐0001‐SuppMat.pdf [file ADBI-10-e00274-s001.pdf]

Supporting Information for

Copper overload affects α-synuclein clearance mechanisms in a Parkinson's disease in vitro model

Debora Musarò, Marina Damato, Chiara Coppola, Marco Greco, Michele Maffia**

Raw data: Beclin-1

Figure 2 A

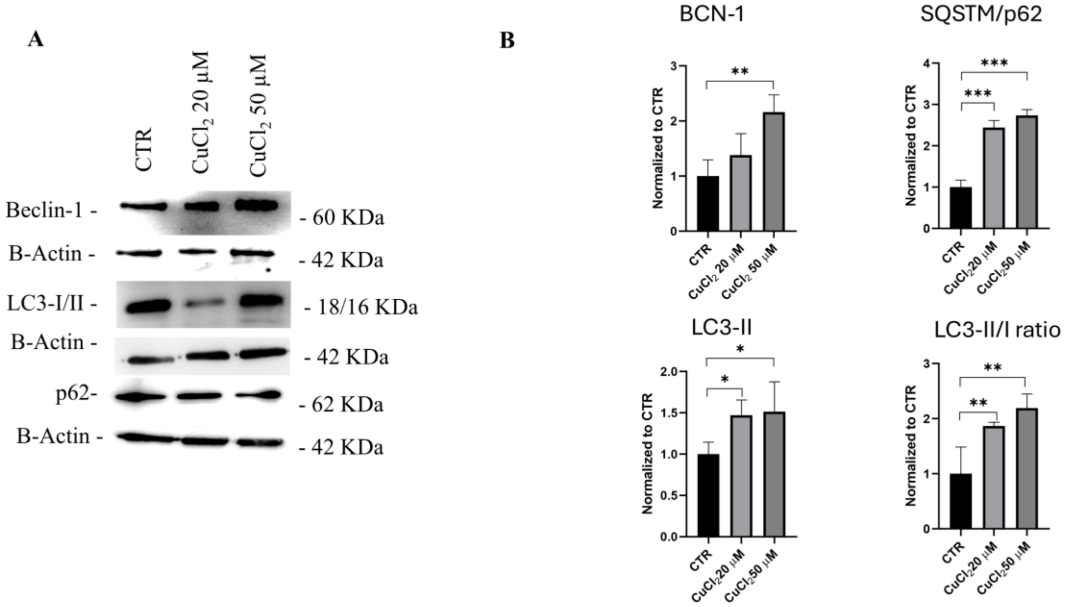

Figure 2, Effects of copper on autophagic markers. Differentiated SH-SY5Y cells were treated with 20 or 50 µM CuCl2 for 48 h. (A) Representative immunoblot showing Beclin-1, LC3-I/II, and SQSTM1/p62 protein levels. (B) Densitometric quantification of Beclin-1, LC3-I/II, LC3-II/I ratio and SQSTM1/p62 levels normalized to β-actin (n = 4). Data are presented as mean ± SD. Statistical significance versus untreated control (CTR) was determined by one-way ANOVA followed by Dunnett's post hoc test (α = 0.05). Analyses were performed using GraphPad Prism 8.0.1. * $p < 0.0332$; ** $p < 0.0021$; *** $p < 0.0002$.

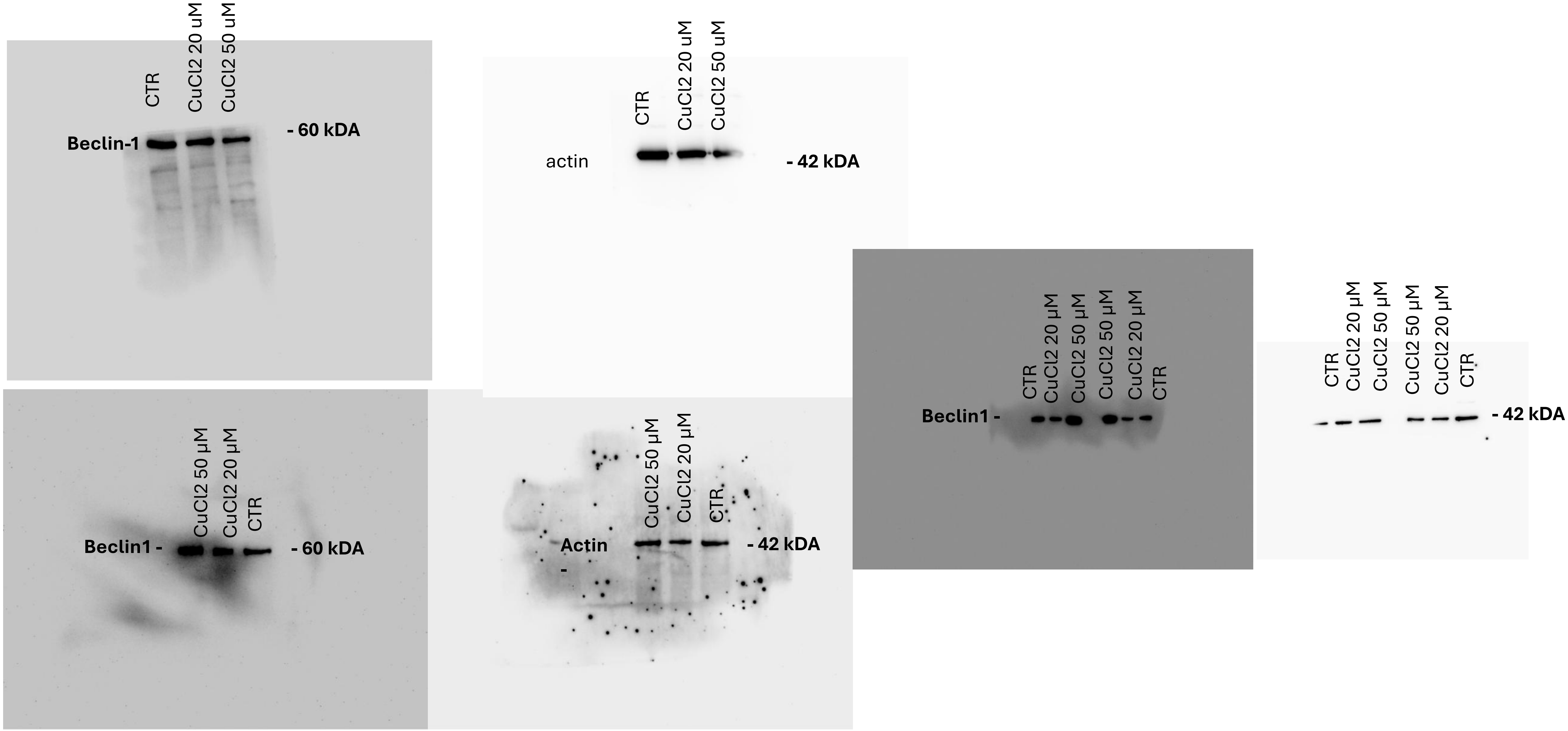
CTR
CuCl2 20 uM
CuCl2 50 uM
Beclin-1
- 60 kDA
CTR
CuCl2 20 uM
CuCl2 50 uM
actin
- 42 kDA
CTR
CuCl2 20 µM
CuCl2 50 µM
CuCl2 50 µM
CuCl2 20 µM
CTR
Beclin1 -
CTR
CuCl2 20 µM
CuCl2 50 µM
CuCl2 50 µM
CuCl2 20 µM
CTR
- 42 kDA
CuCl2 50 µM
CuCl2 20 µM
CTR
Beclin1 -
- 60 kDA
CuCl2 50 µM
CuCl2 20 µM
CTR
Actin
-
- 42 kDA

Raw data: LC3-I/II; p62

Figure 2 A, B
Figure 3 A, B
Figure 4 A, B

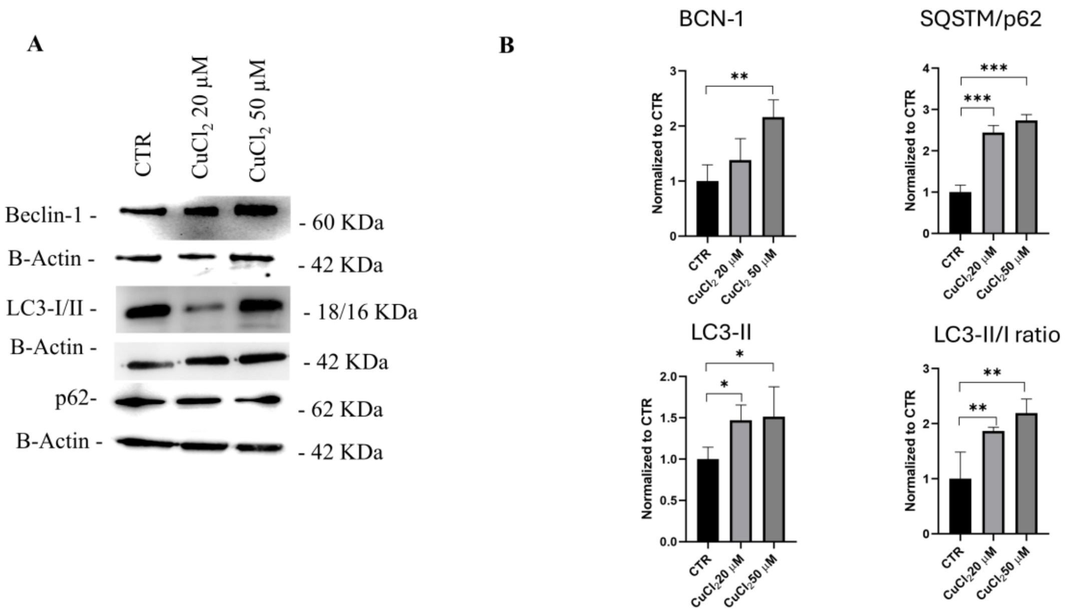

Figure 2. Effects of copper on autophagic markers. Differentiated SH-SY5Y cells were treated with 20 or 50 µM CuCl2 for 48 h. (A) Representative immunoblot showing Beclin-1, LC3-I/II, and SQSTM1/p62 protein levels. (B) Densitometric quantification of Beclin-1, LC3-I/II, LC3-II/I ratio and SQSTM1/p62 levels normalized to β-actin (n = 4). Data are presented as mean ± SD. Statistical significance versus untreated control (CTR) was determined by one-way ANOVA followed by Dunnett's post hoc test (α = 0.05). Analyses were performed using GraphPad Prism 8.0.1. * $p < 0.0332$; ** $p < 0.0021$; *** $p < 0.0002$.

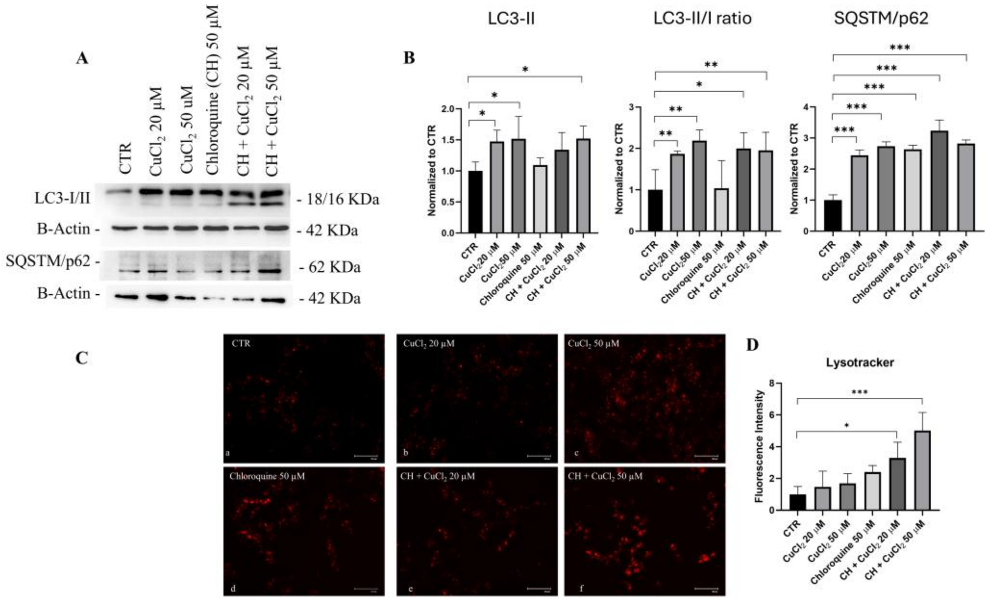

Figure 3, Copper induces autophagic flux blockage. Differentiated SH-SY5Y cells were treated with 20 or 50 µM CuCl2 for 48 h in the absence or presence of chloroquine (CH, 50 µM, 1 h). (A) Representative immunoblot showing LC3-I/II and SQSTM1/p62 protein levels. (B) Densitometric quantification of LC3-I/II, LC3-II/I ratio, and SQSTM1/p62 levels normalized to β-actin (n = 4). Data are presented as mean ± SD. Statistical significance versus untreated control (CTR) was determined by one-way ANOVA followed by Dunnett's post hoc test (α = 0.05). Analyses were performed using GraphPad Prism 8.0.1. * $p < 0.0332$; ** $p < 0.0021$; *** $p < 0.0002$. (C) Representative fluorescence microscopy images (a–f) of cells stained with LysoTracker Red DND-99 to mark lysosomes (red). Scale bar, 125 µm; magnification, 20×. (D) Graphical representation of the fluorescence intensity ratio LysoTracker Red DND-99 normalized to CTR (n = 3).

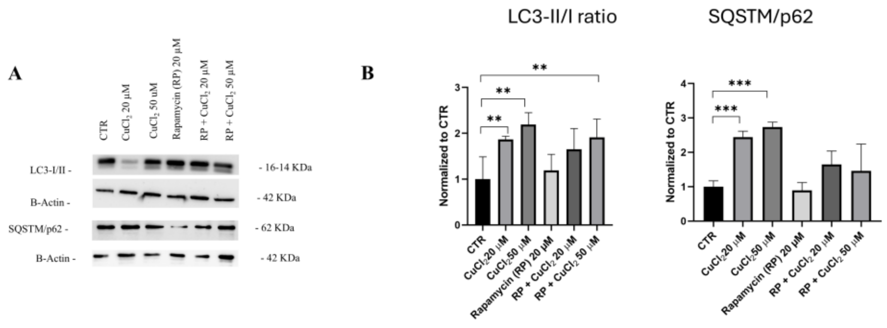

Figure 4. Rapamycin rescues autophagic flux. Differentiated SH-SY5Y cells were treated with 20 or 50 µM CuCl2 for 48 h in the presence or absence of rapamycin (RP, 20 µM, 1 h). (A) Representative immunoblot showing LC3-I/II and SQSTM1/p62 protein levels. (B) Densitometric quantification of the LC3-II/I ratio and SQSTM1/p62 levels normalized to β-actin (n = 4). Data are presented as mean ± SD. Statistical significance was assessed by one-way ANOVA followed by Dunnett's post hoc test (α = 0.05). Analyses were performed using GraphPad Prism 8.0.1. * $p < 0.0332$; ** $p < 0.0021$; *** $p < 0.0002$ versus untreated control (CTR).

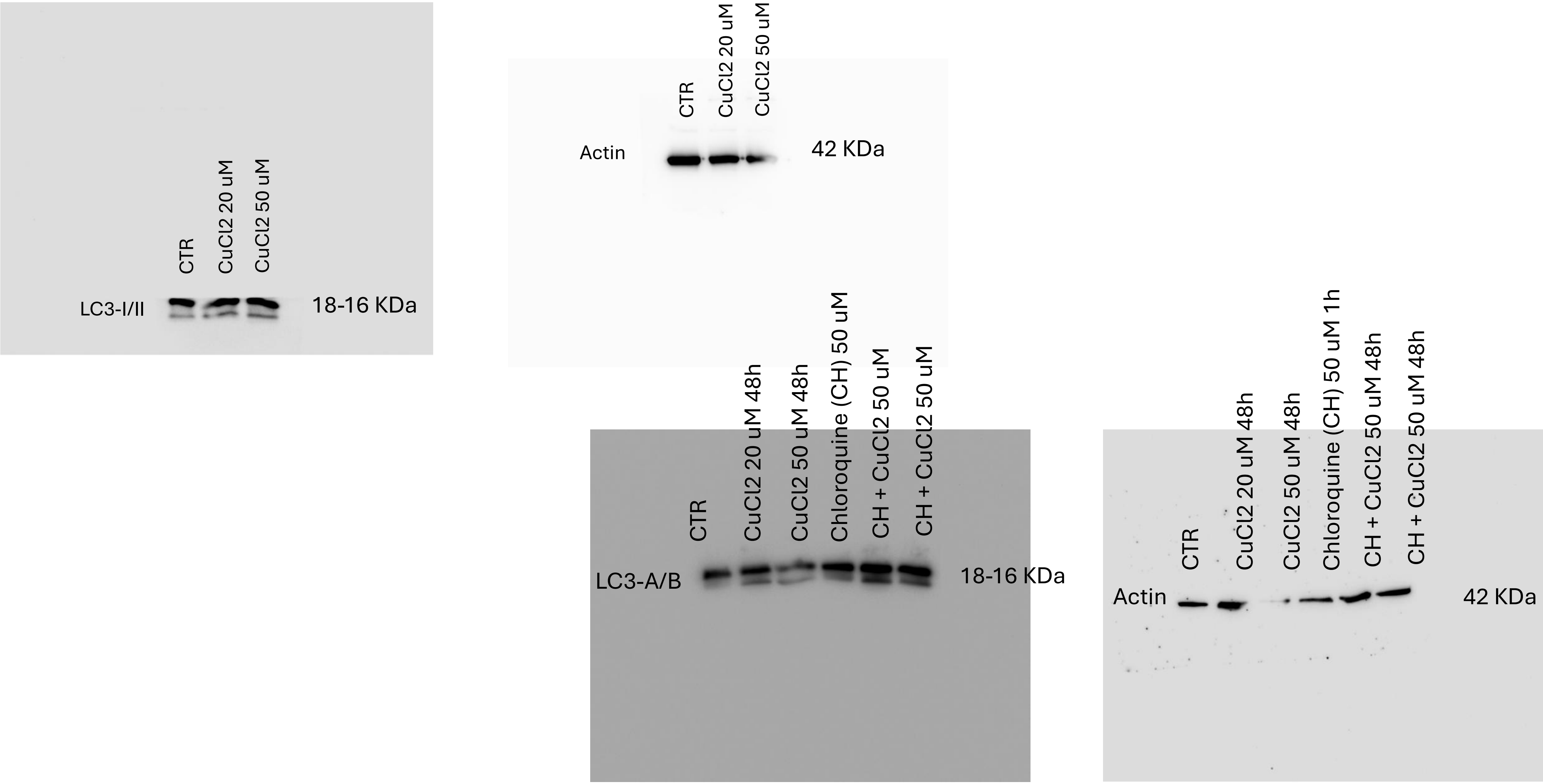
CTR
CuCl2 20 uM
CuCl2 50 uM
LC3-I/II
18-16 KDa
CTR
CuCl2 20 uM
CuCl2 50 uM
Actin
42 KDa
CTR
CuCl2 20 uM 48h
CuCl2 50 uM 48h
Chloroquine (CH) 50 uM
CH + CuCl2 50 uM
CH + CuCl2 50 uM
LC3-A/B
18-16 KDa
CTR
CuCl2 20 uM 48h
CuCl2 50 uM 48h
Chloroquine (CH) 50 uM 1h
CH + CuCl2 50 uM 48h
CH + CuCl2 50 uM 48h
Actin
42 KDa

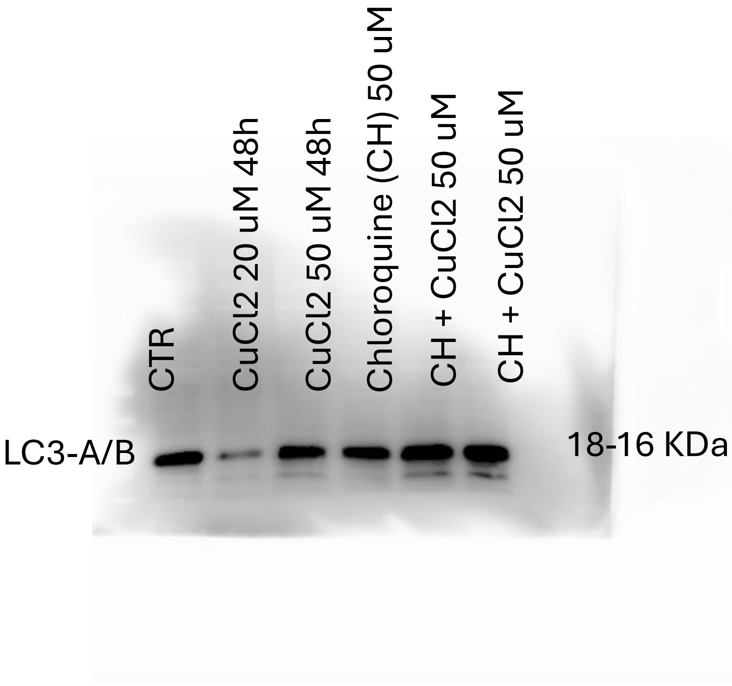
CTR
CuCl2 20 uM 48h
CuCl2 50 uM 48h
Chloroquine (CH) 50 uM
CH + CuCl2 50 uM
CH + CuCl2 50 uM
LC3-A/B
18-16 KDa

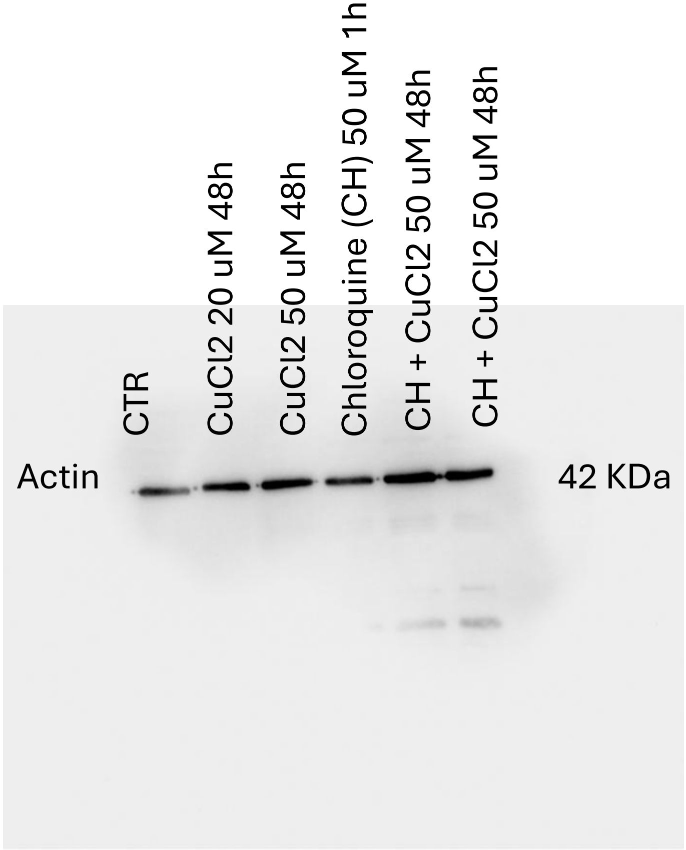
CTR
CuCl2 20 uM 48h
CuCl2 50 uM 48h
Chloroquine (CH) 50 uM 1h
CH + CuCl2 50 uM 48h
CH + CuCl2 50 uM 48h
Actin
42 KDa

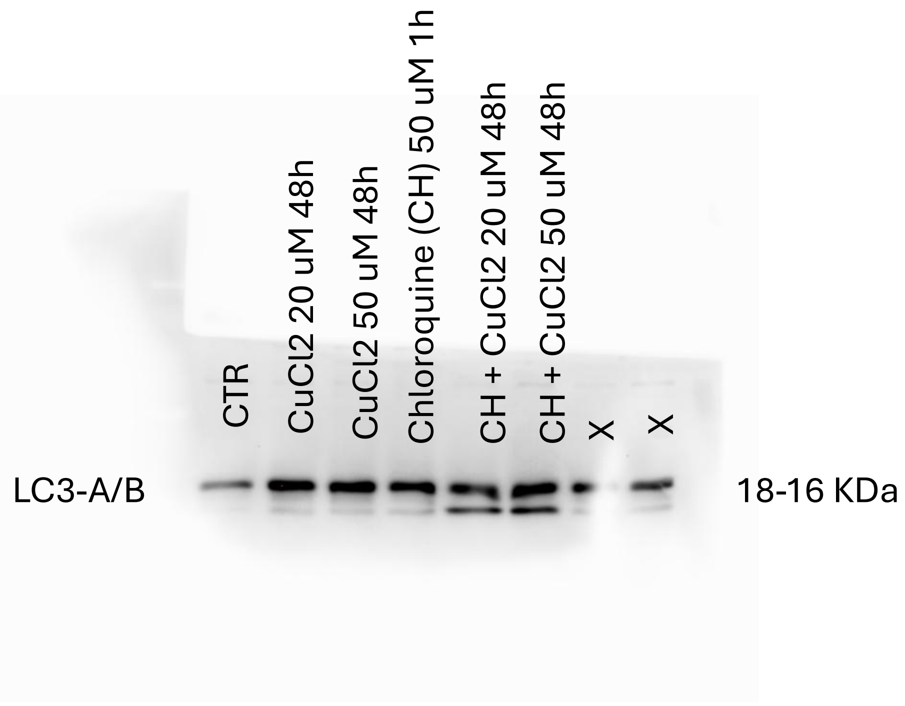
CTR
CuCl2 20 uM 48h
CuCl2 50 uM 48h
Chloroquine (CH) 50 uM 1h
CH + CuCl2 20 uM 48h
CH + CuCl2 50 uM 48h
X
X
LC3-A/B
18-16 KDa

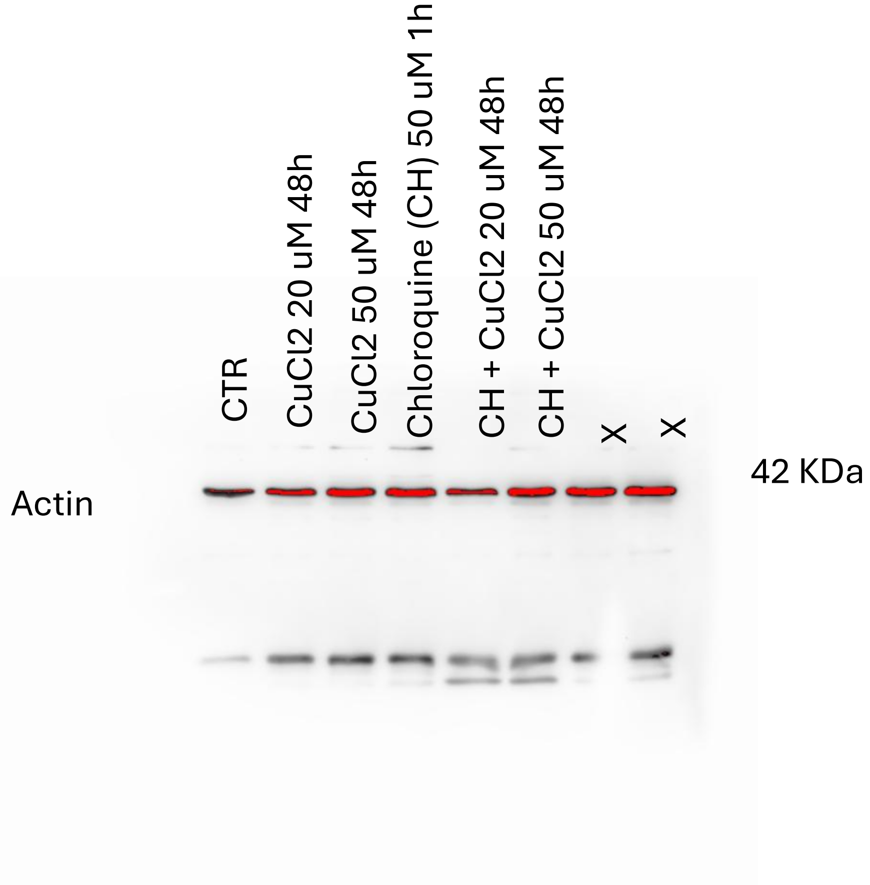
CTR
CuCl2 20 uM 48h
CuCl2 50 uM 48h
Chloroquine (CH) 50 uM 1h
CH + CuCl2 20 uM 48h
CH + CuCl2 50 uM 48h
X
X
Actin
42 KDa

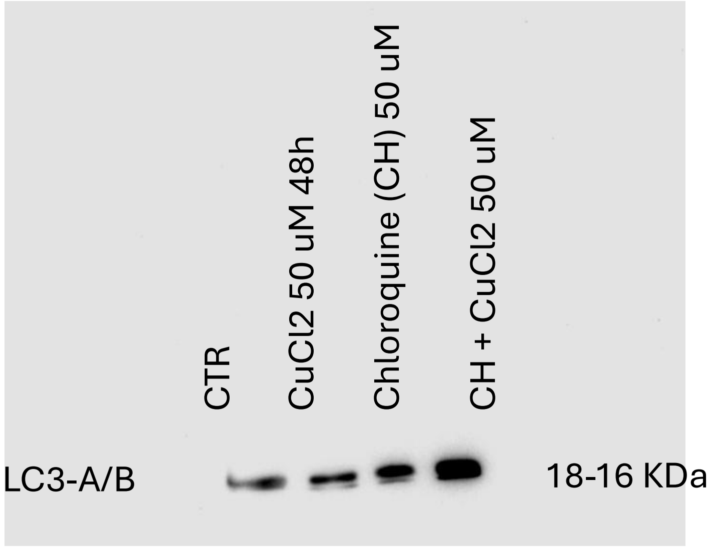
CTR
CuCl2 50 uM 48h
Chloroquine (CH) 50 uM
CH + CuCl2 50 uM
LC3-A/B
18-16 KDa

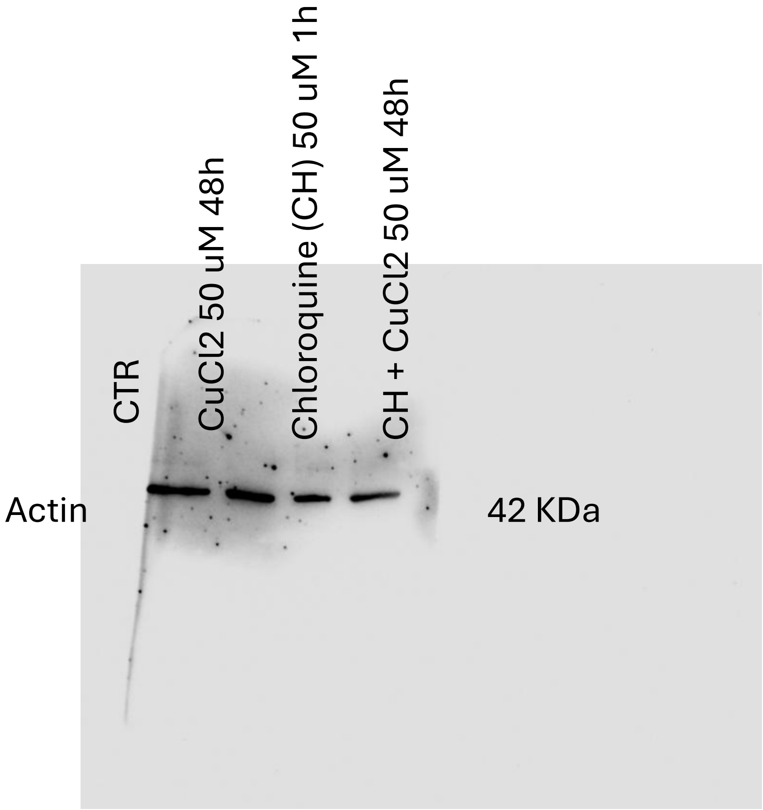
CTR
CuCl2 50 uM 48h
Chloroquine (CH) 50 uM 1h
CH + CuCl2 50 uM 48h
Actin
42 KDa

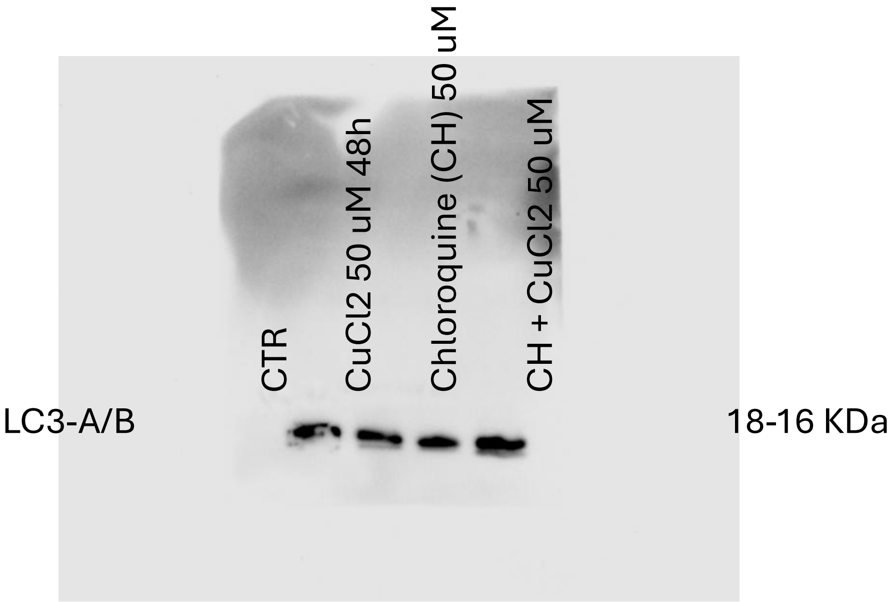
CTR
CuCl2 50 uM 48h
Chloroquine (CH) 50 uM
CH + CuCl2 50 uM
LC3-A/B
18-16 KDa

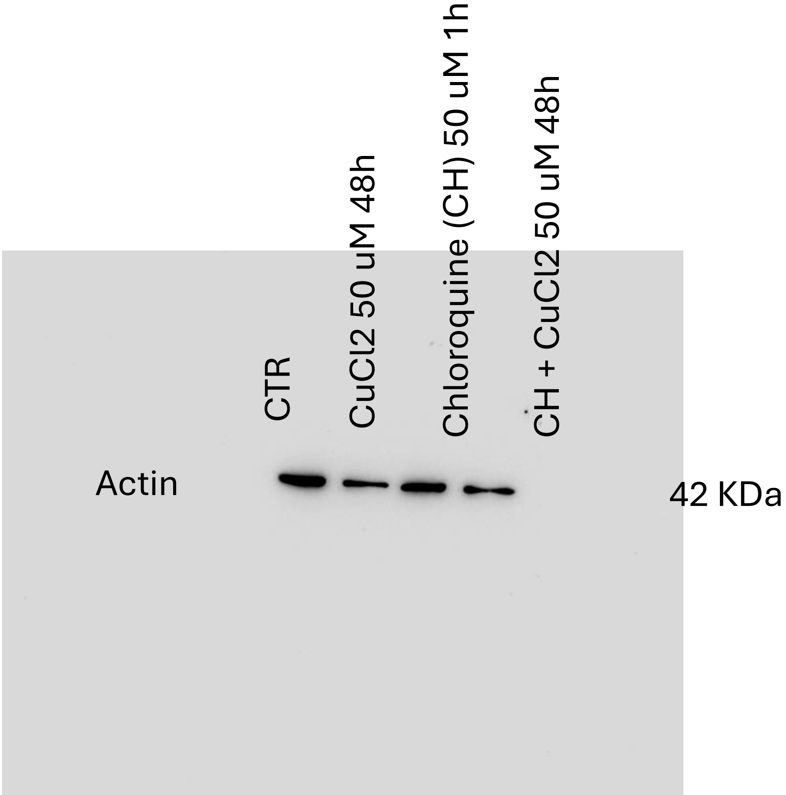
CTR
CuCl2 50 uM 48h
Chloroquine (CH) 50 uM 1h
CH + CuCl2 50 uM 48h
Actin
42 KDa

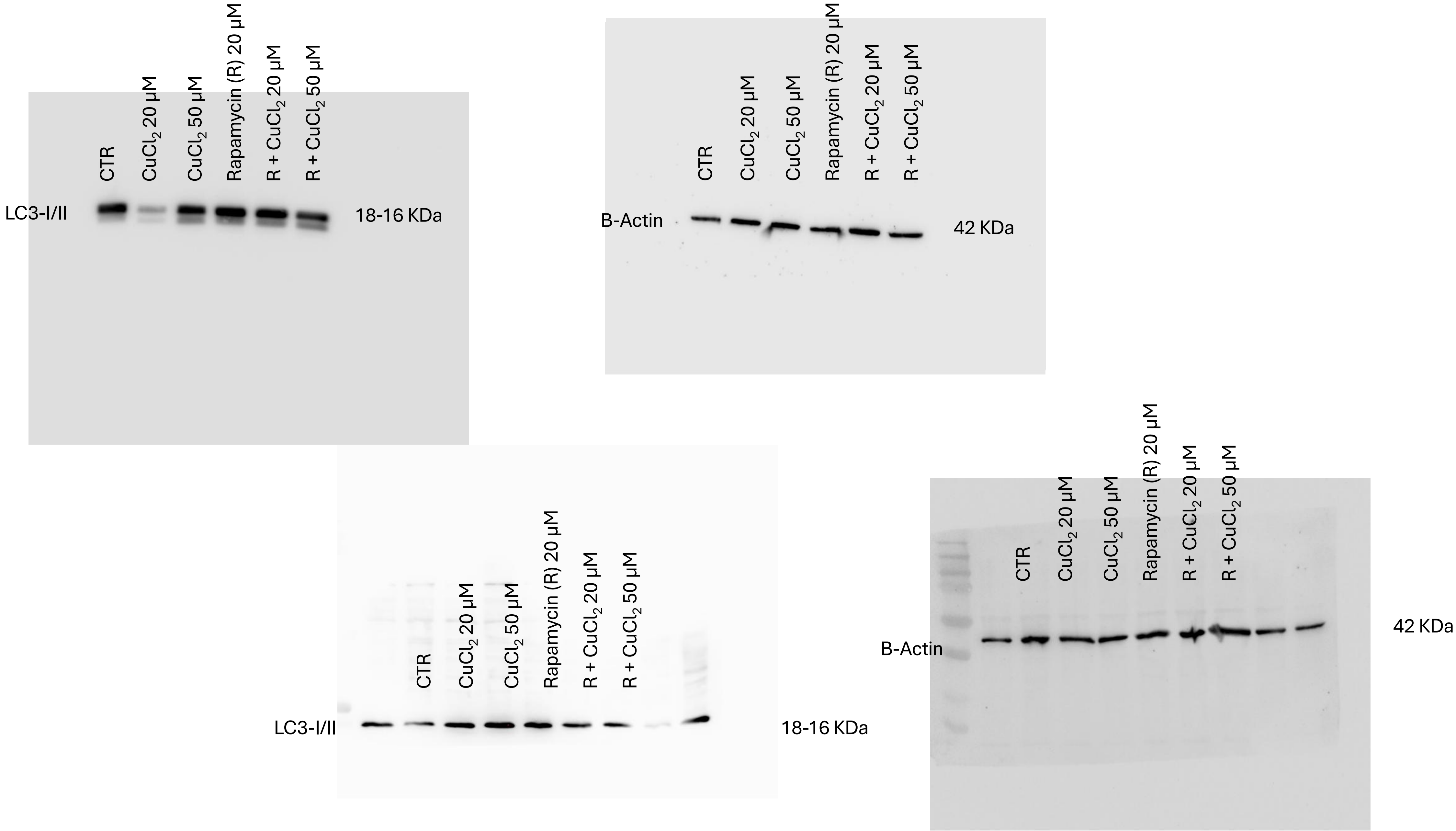

CTR
CuCl$_2$ 20 µM
CuCl$_2$ 50 µM
Rapamycin (R) 20 µM
R + CuCl$_2$ 20 µM
R + CuCl$_2$ 50 µM
LC3-I/II
18-16 KDa
CTR
CuCl$_2$ 20 µM
CuCl$_2$ 50 µM
Rapamycin (R) 20 µM
R + CuCl$_2$ 20 µM
R + CuCl$_2$ 50 µM
B-Actin
42 KDa
CTR
CuCl$_2$ 20 µM
CuCl$_2$ 50 µM
Rapamycin (R) 20 µM
R + CuCl$_2$ 20 µM
R + CuCl$_2$ 50 µM
LC3-I/II
18-16 KDa
CTR
CuCl$_2$ 20 µM
CuCl$_2$ 50 µM
Rapamycin (R) 20 µM
R + CuCl$_2$ 20 µM
R + CuCl$_2$ 50 µM
B-Actin
42 KDa

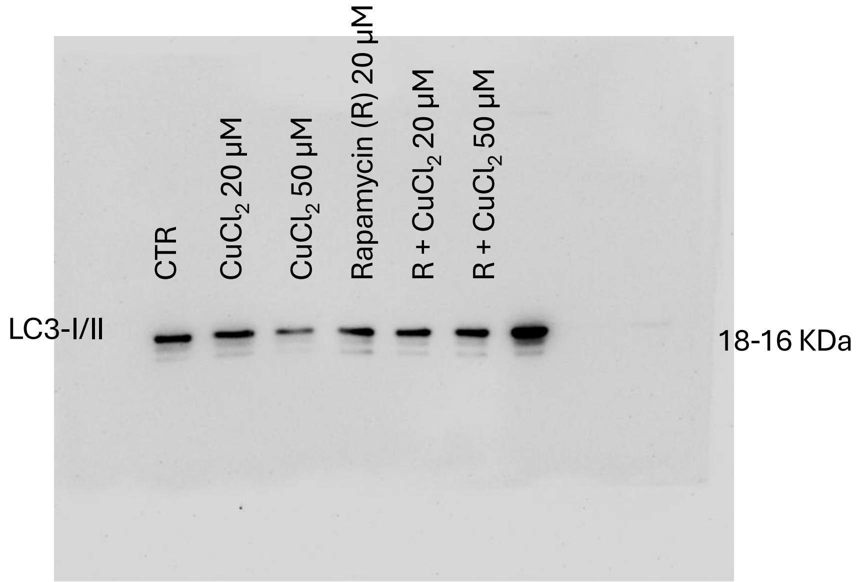
CTR
$CuCl_2$ 20 µM
$CuCl_2$ 50 µM
Rapamycin (R) 20 µM
R + $CuCl_2$ 20 µM
R + $CuCl_2$ 50 µM
LC3-I/II
18-16 KDa

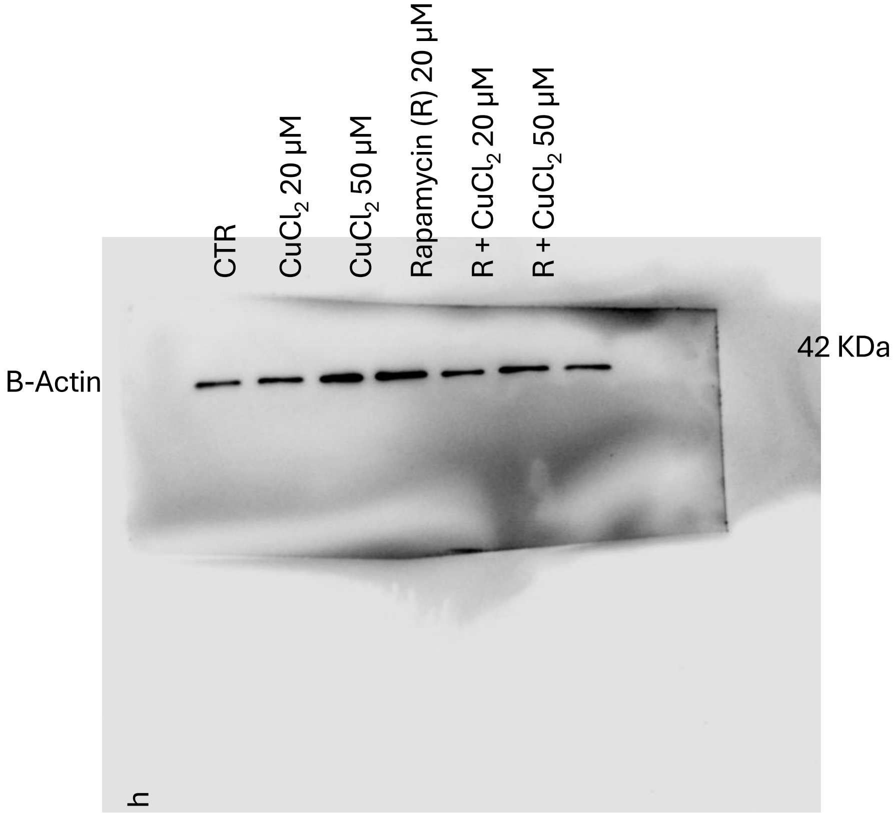
CTR
$CuCl_2$ 20 µM
$CuCl_2$ 50 µM
Rapamycin (R) 20 µM
R + $CuCl_2$ 20 µM
R + $CuCl_2$ 50 µM
B-Actin
42 KDa

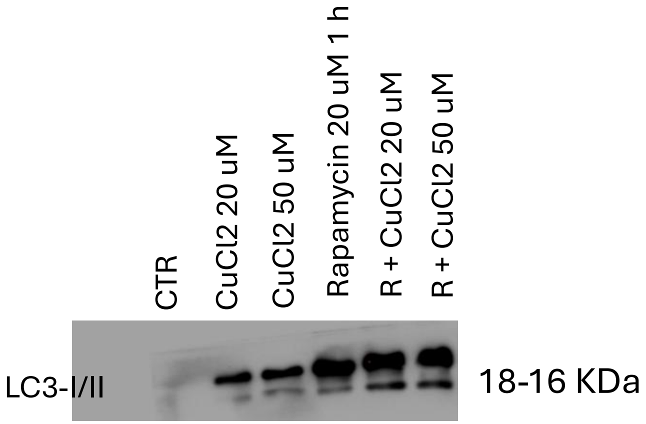
CTR
CuCl2 20 uM
CuCl2 50 uM
Rapamycin 20 uM 1 h
R + CuCl2 20 uM
R + CuCl2 50 uM
LC3-I/II
18-16 KDa

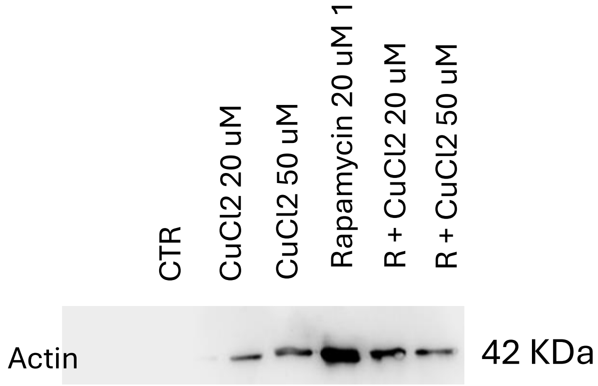
CTR
CuCl2 20 uM
CuCl2 50 uM
Rapamycin 20 uM 1 h
R + CuCl2 20 uM
R + CuCl2 50 uM
Actin
42 KDa

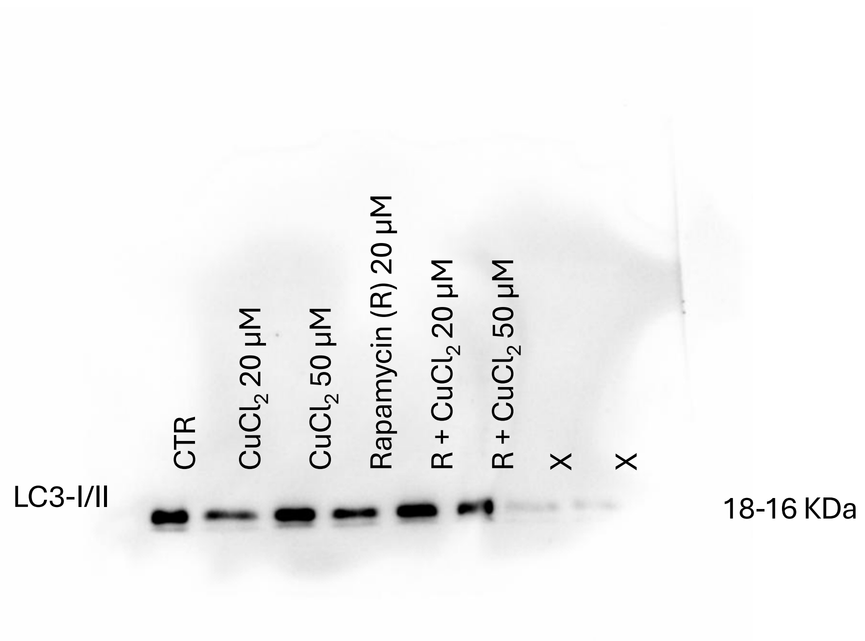
CTR
CuCl2 20 µM
CuCl2 50 µM
Rapamycin (R) 20 µM
R + CuCl2 20 µM
R + CuCl2 50 µM
X
X
LC3-I/II
18-16 KDa

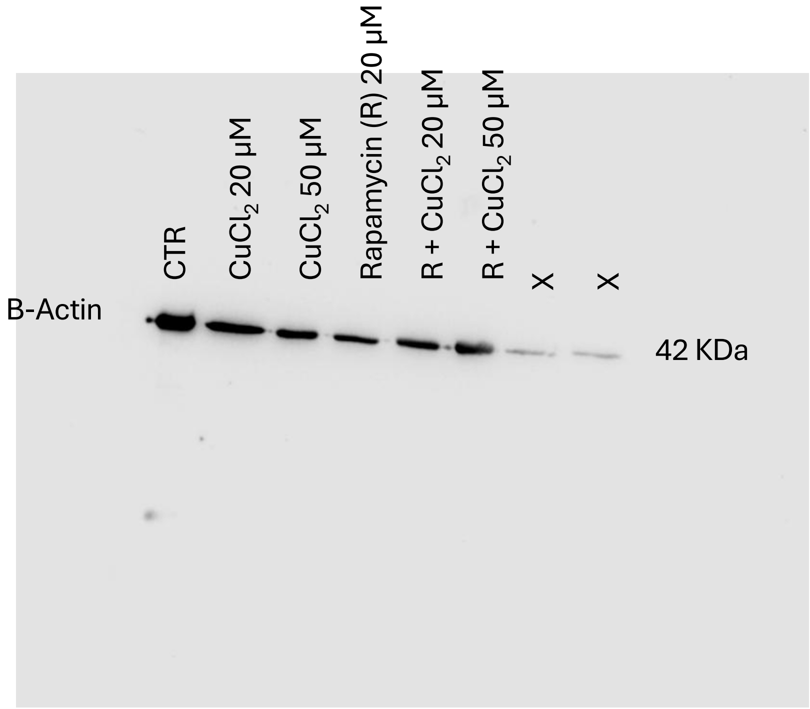
CTR
CuCl2 20 µM
CuCl2 50 µM
Rapamycin (R) 20 µM
R + CuCl2 20 µM
R + CuCl2 50 µM
X
X
B-Actin
42 KDa

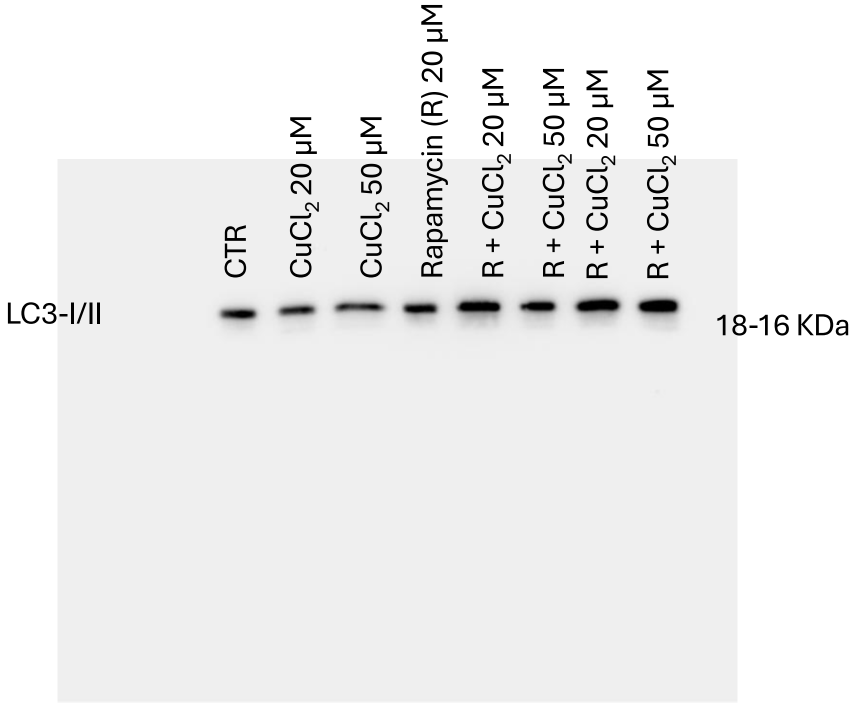
CTR
CuCl$_2$ 20 μM
CuCl$_2$ 50 μM
Rapamycin (R) 20 μM
R + CuCl$_2$ 20 μM
R + CuCl$_2$ 50 μM
R + CuCl$_2$ 20 μM
R + CuCl$_2$ 50 μM
LC3-I/II
18-16 KDa

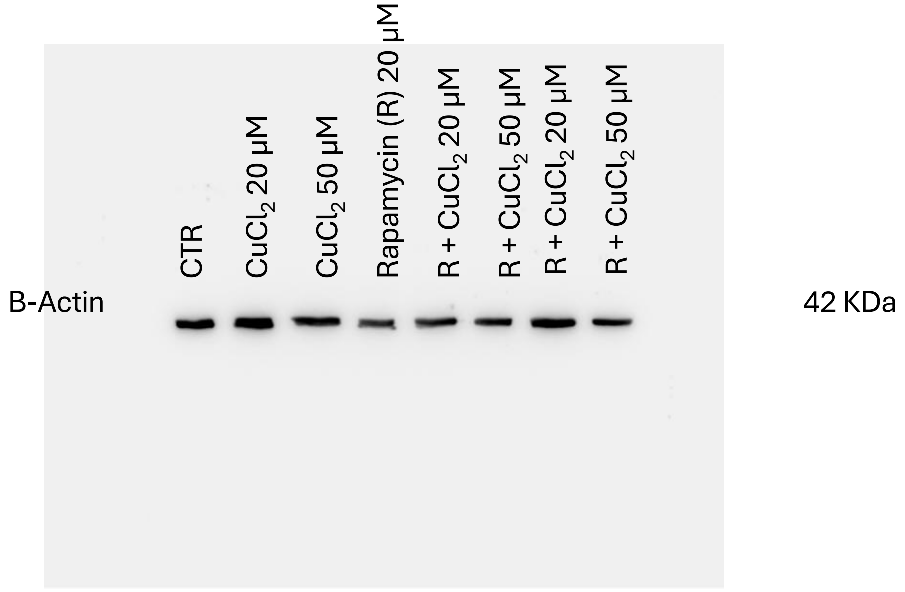
CTR
CuCl$_2$ 20 μM
CuCl$_2$ 50 μM
Rapamycin (R) 20 μM
R + CuCl$_2$ 20 μM
R + CuCl$_2$ 50 μM
R + CuCl$_2$ 20 μM
R + CuCl$_2$ 50 μM
B-Actin
42 KDa

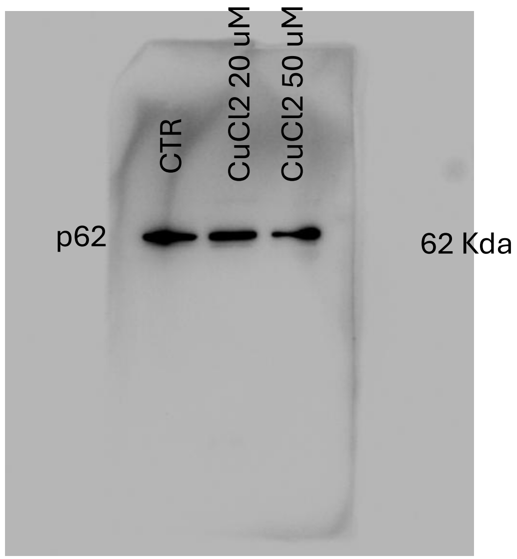
CTR
CuCl2 20 uM
CuCl2 50 uM
p62
62 Kda

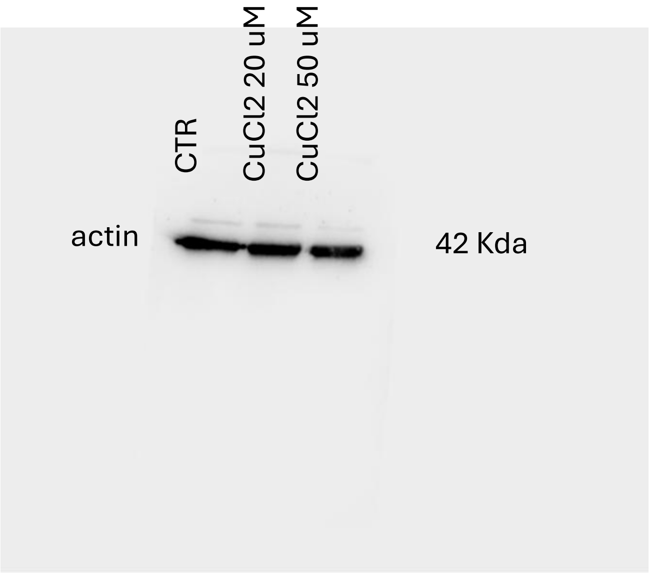
CTR
CuCl2 20 uM
CuCl2 50 uM
actin
42 Kda

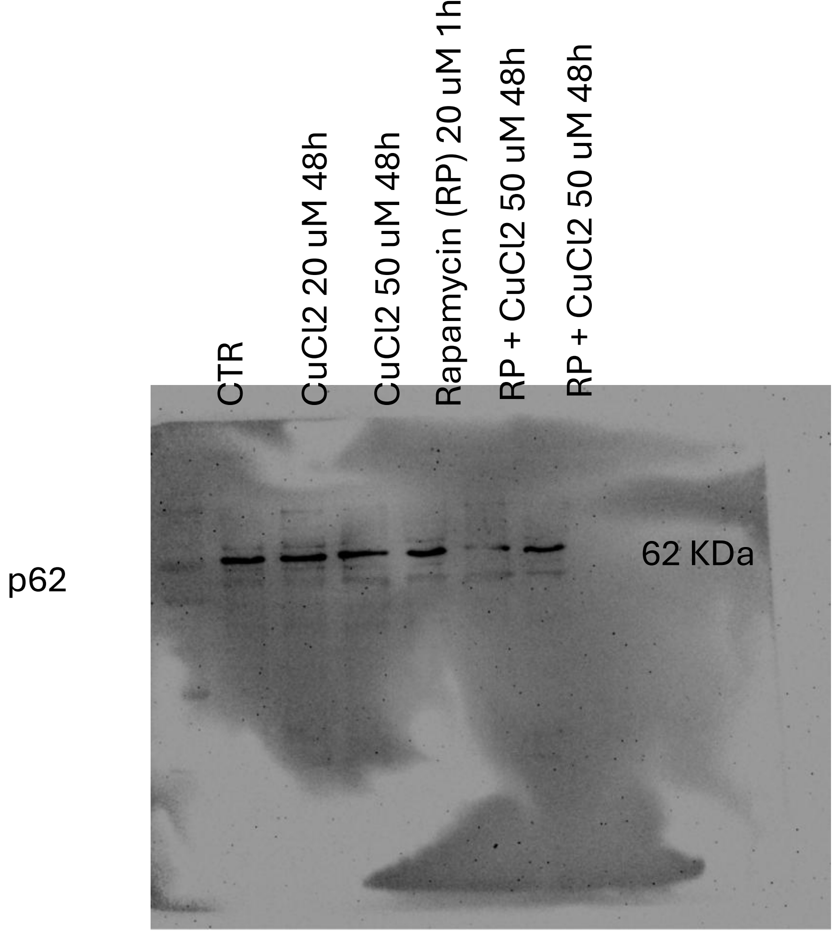
CTR
CuCl2 20 uM 48h
CuCl2 50 uM 48h
Rapamycin (RP) 20 uM 1h
RP + CuCl2 50 uM 48h
RP + CuCl2 50 uM 48h
p62
62 KDa

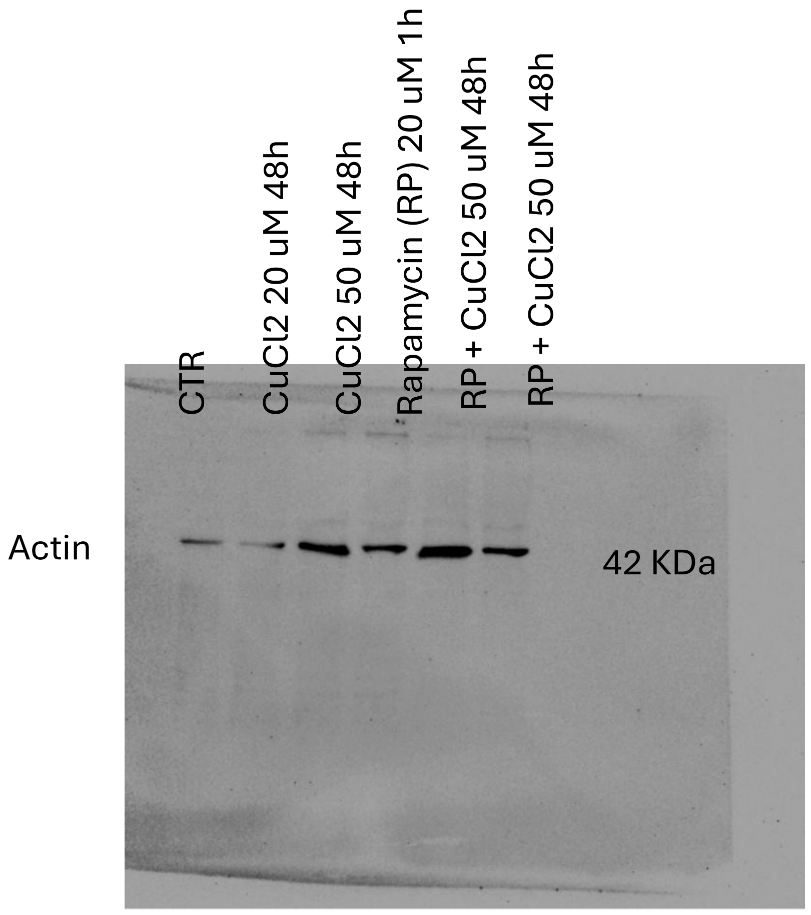
CTR
CuCl2 20 uM 48h
CuCl2 50 uM 48h
Rapamycin (RP) 20 uM 1h
RP + CuCl2 50 uM 48h
RP + CuCl2 50 uM 48h
Actin
42 KDa

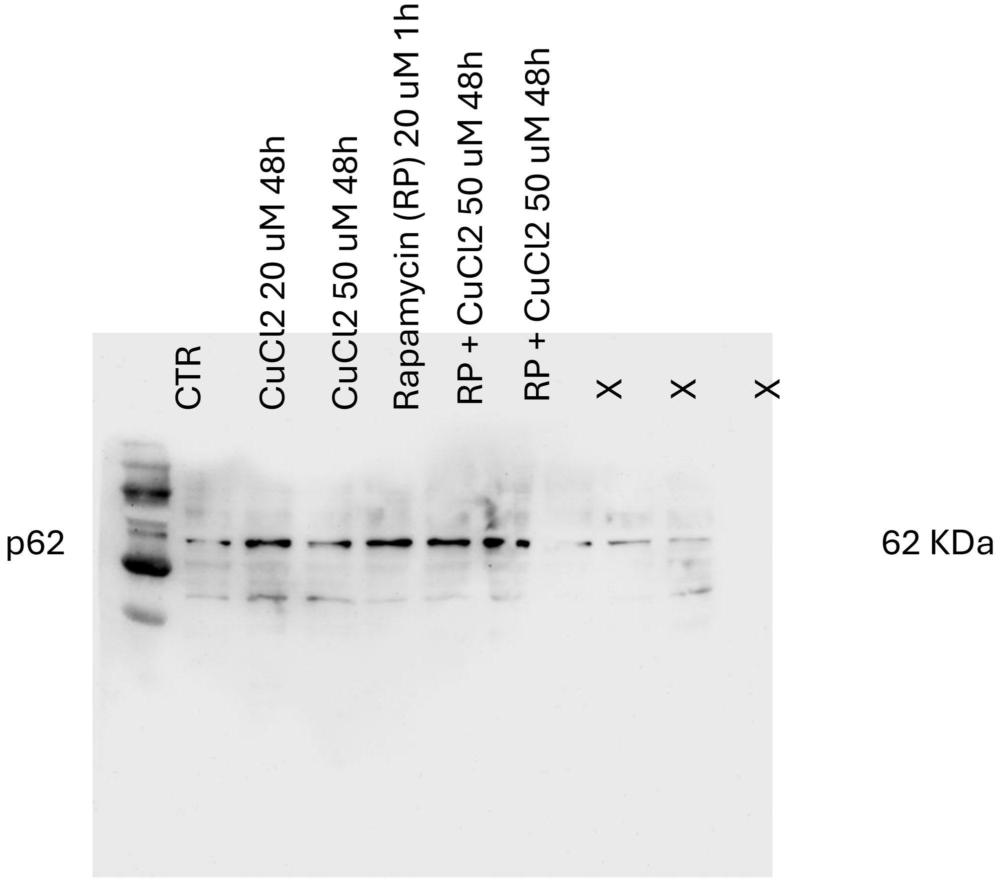
CTR
CuCl2 20 uM 48h
CuCl2 50 uM 48h
Rapamycin (RP) 20 uM 1h
RP + CuCl2 50 uM 48h
RP + CuCl2 50 uM 48h
X
X
X
p62
62 KDa

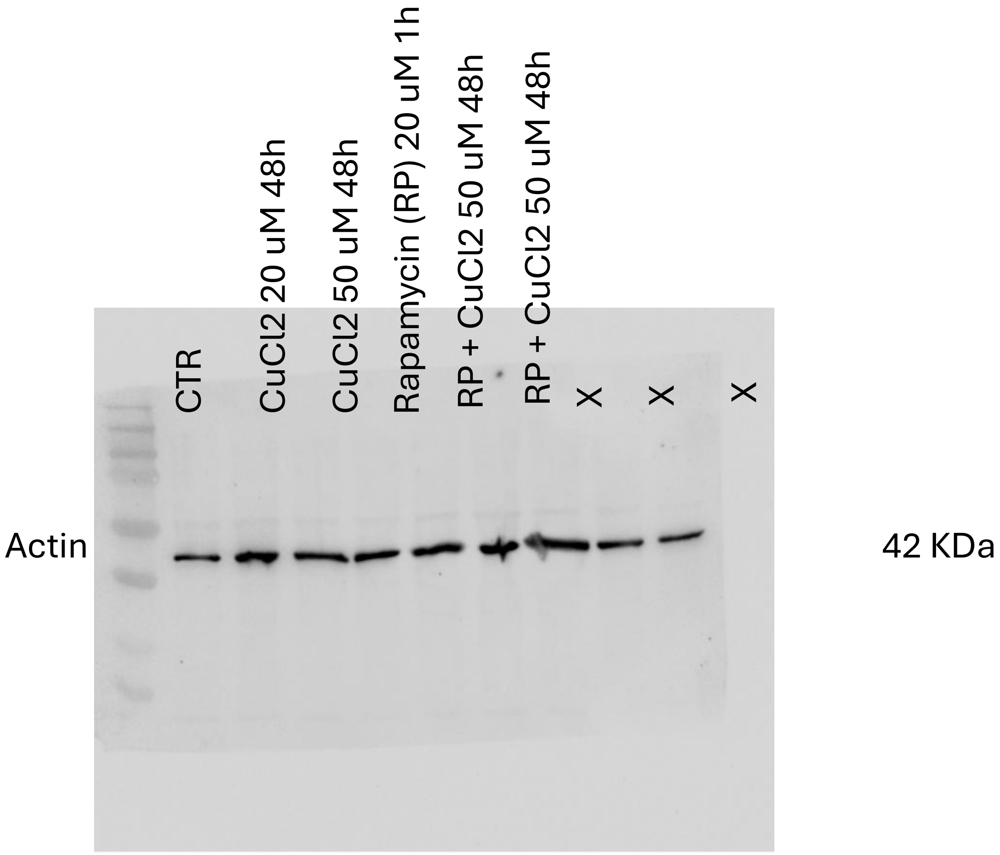
CTR
CuCl2 20 uM 48h
CuCl2 50 uM 48h
Rapamycin (RP) 20 uM 1h
RP + CuCl2 50 uM 48h
RP + CuCl2 50 uM 48h
X
X
X
Actin
42 KDa

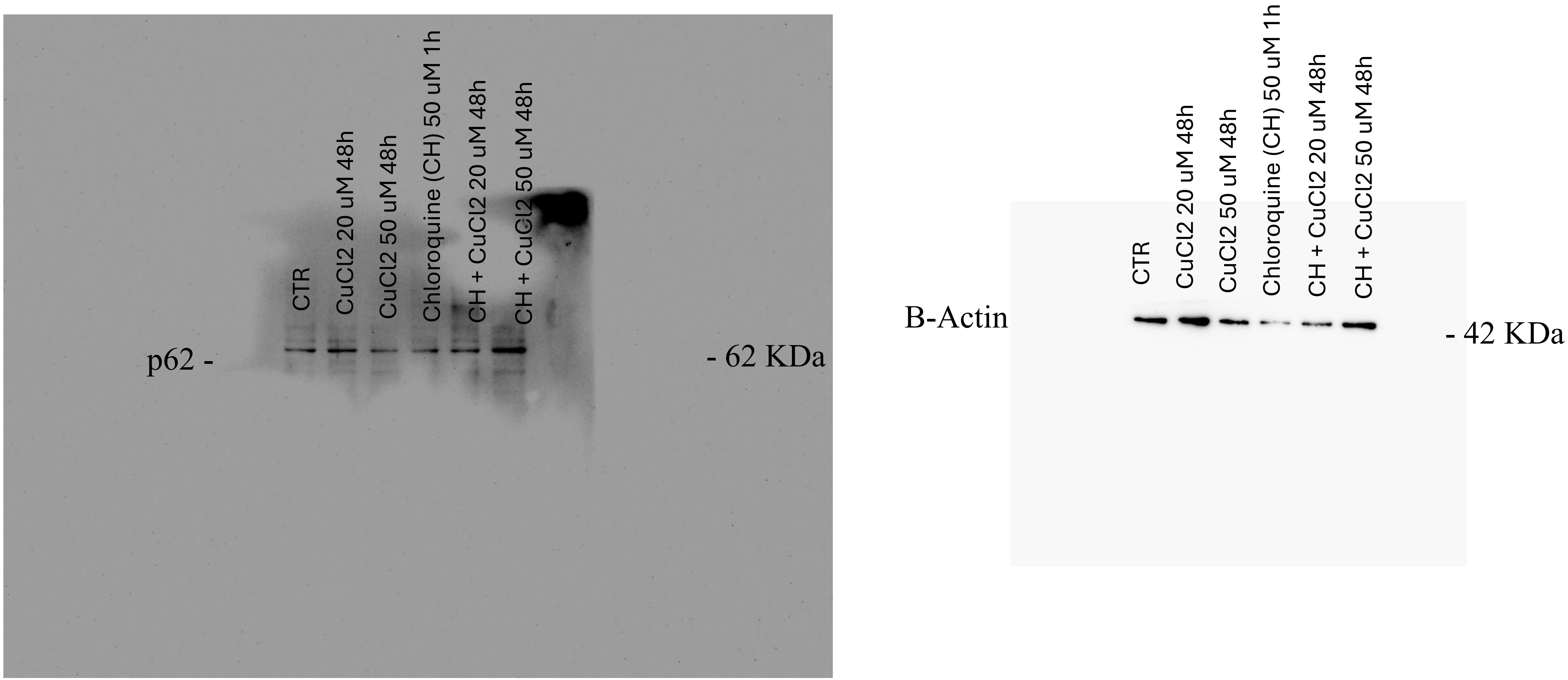

CTR
CuCl2 20 uM 48h
CuCl2 50 uM 48h
Chloroquine (CH) 50 uM 1h
CH + CuCl2 20 uM 48h
CH + CuCl2 50 uM 48h
p62 -
- 62 KDa
CTR
CuCl2 20 uM 48h
CuCl2 50 uM 48h
Chloroquine (CH) 50 uM 1h
CH + CuCl2 20 uM 48h
CH + CuCl2 50 uM 48h
B-Actin
- 42 KDa

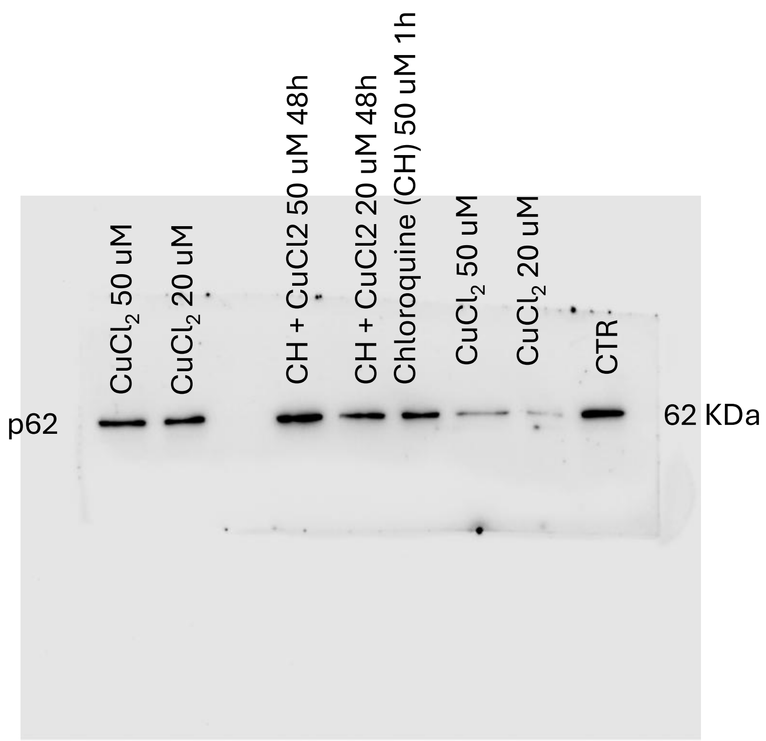

CuCl2 50 uM
CuCl2 20 uM
CH + CuCl2 50 uM 48h
CH + CuCl2 20 uM 48h
Chloroquine (CH) 50 uM 1h
CuCl2 50 uM
CuCl2 20 uM
CTR
p62
62 KDa

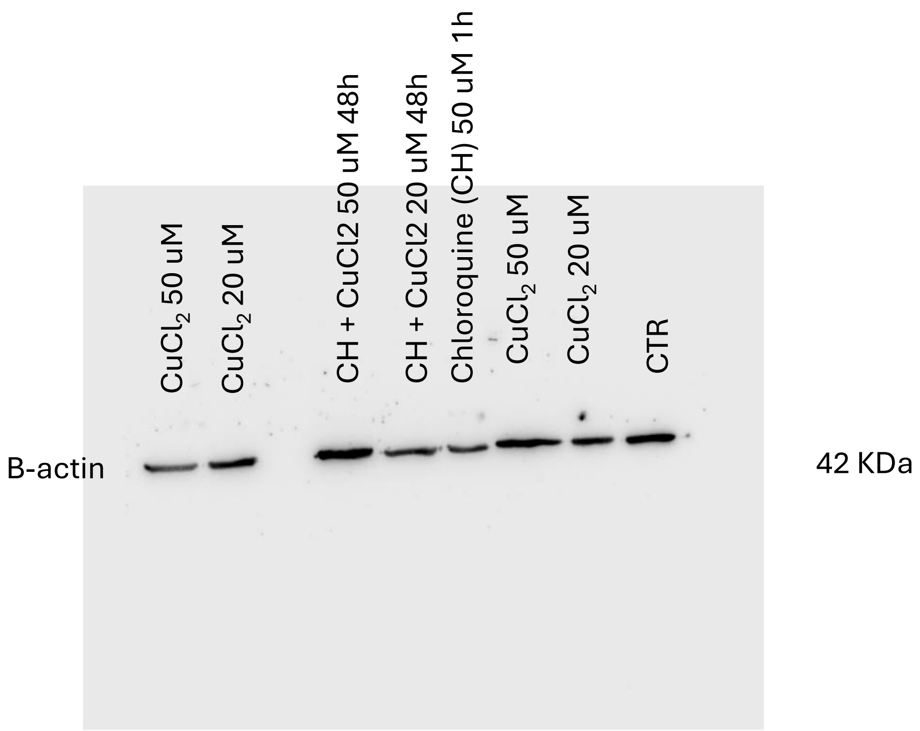

CuCl2 50 uM
CuCl2 20 uM
CH + CuCl2 50 uM 48h
CH + CuCl2 20 uM 48h
Chloroquine (CH) 50 uM 1h
CuCl2 50 uM
CuCl2 20 uM
CTR
B-actin
42 KDa

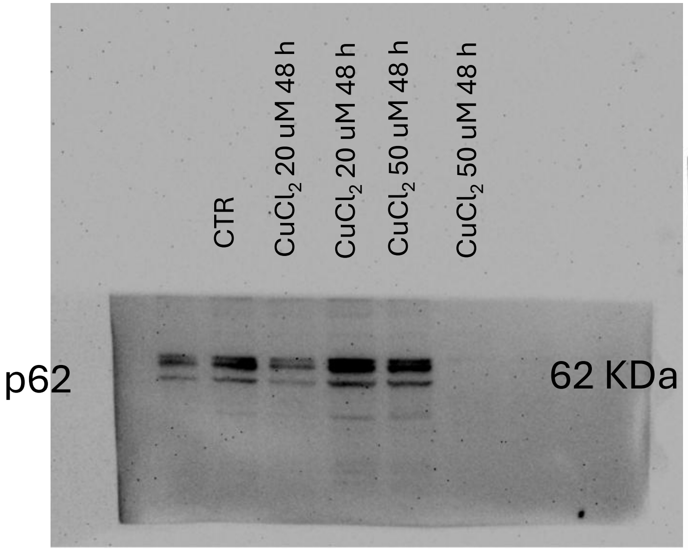
CTR
CuCl2 20 uM 48 h
CuCl2 20 uM 48 h
CuCl2 50 uM 48 h
CuCl2 50 uM 48 h
p62
62 KDa

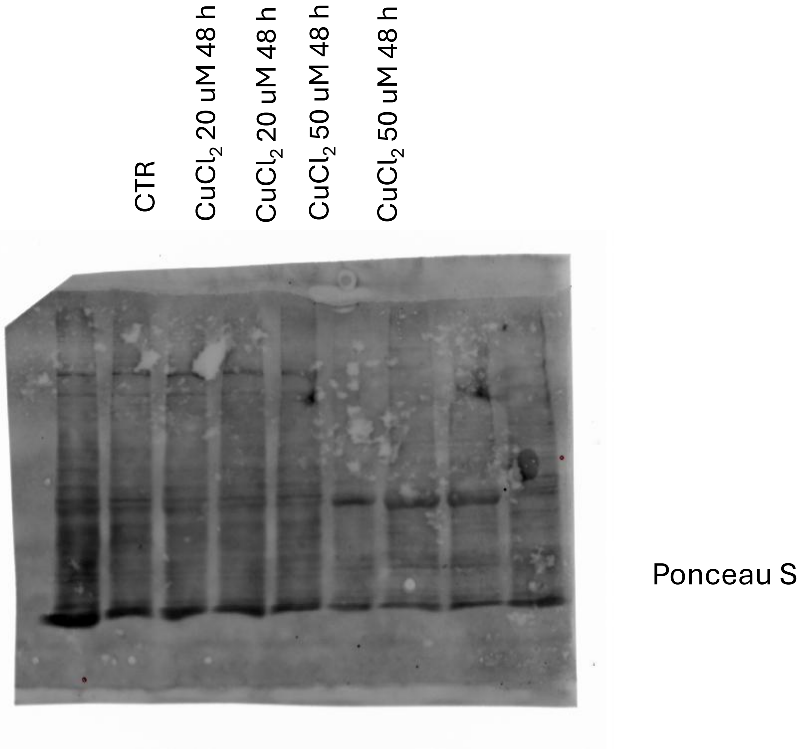
CTR
CuCl2 20 uM 48 h
CuCl2 20 uM 48 h
CuCl2 50 uM 48 h
CuCl2 50 uM 48 h
Ponceau S

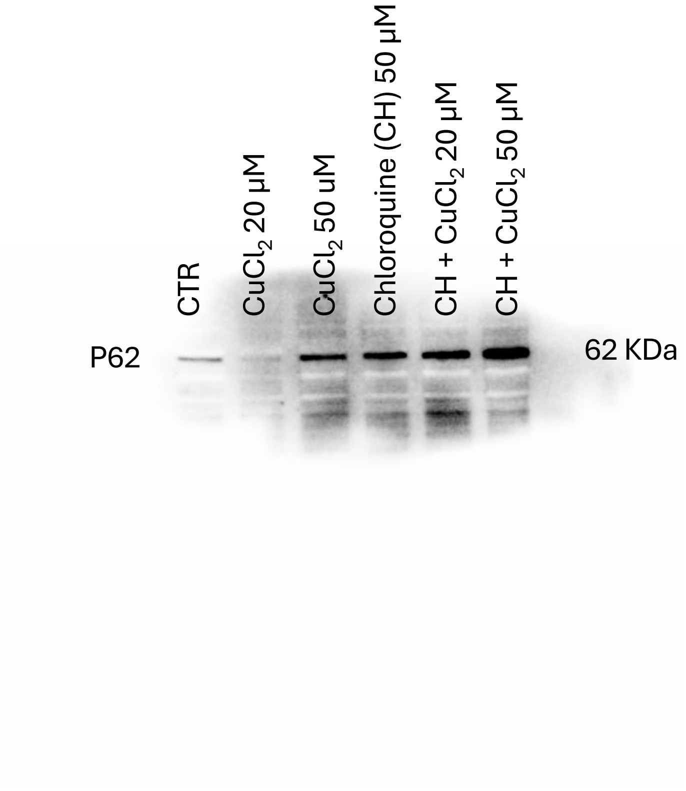
CTR
CuCl2 20 μM
CuCl2 50 uM
Chloroquine (CH) 50 μM
CH + CuCl2 20 μM
CH + CuCl2 50 μM
P62
62 KDa

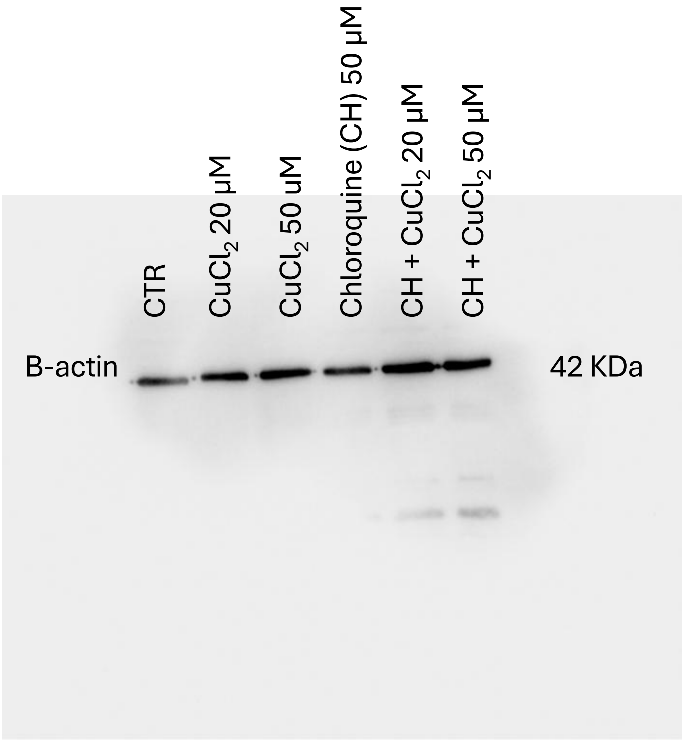
CTR
CuCl2 20 μM
CuCl2 50 uM
Chloroquine (CH) 50 μM
CH + CuCl2 20 μM
CH + CuCl2 50 μM
B-actin
42 KDa

Raw data: Ubiquitin

Figure 5 A, B

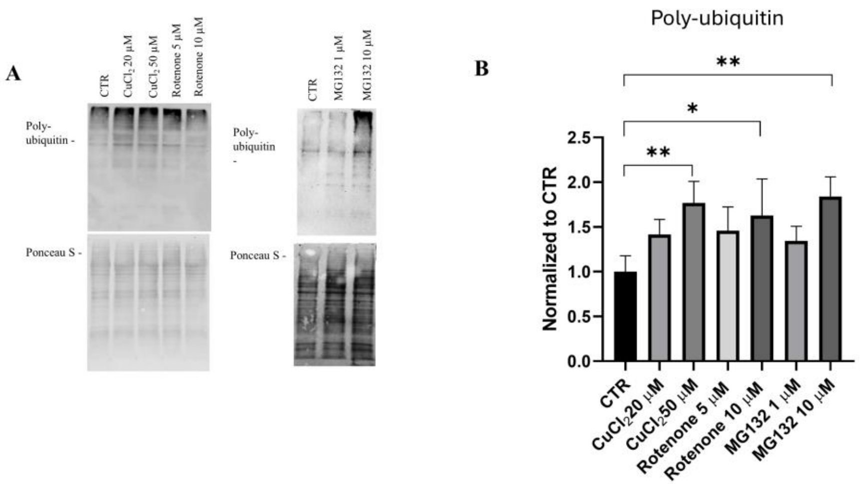

Figure 5, Polyubiquitin accumulation after CuCl2, rotenone, and MG132 treatments. Cells were treated with 20 or 50 µM CuCl2 for 48 h, or with 5 or 10 µM rotenone for 24 h. Cells were also treated with 1 or 10 µM MG132 for 1 h. (A) Representative immunoblot showing polyubiquitinated protein levels. (B) Densitometric quantification of polyubiquitinated proteins normalized to total protein (Ponceau S stain; n = 3). Data are mean ± SD. Statistical significance versus untreated control (CTR) was assessed by one-way ANOVA followed by Tukey's post-hoc test ($\alpha = 0.05$). Analyses were performed using GraphPad Prism 8.0.1. * $p < 0.0332$; ** $p < 0.0021$.

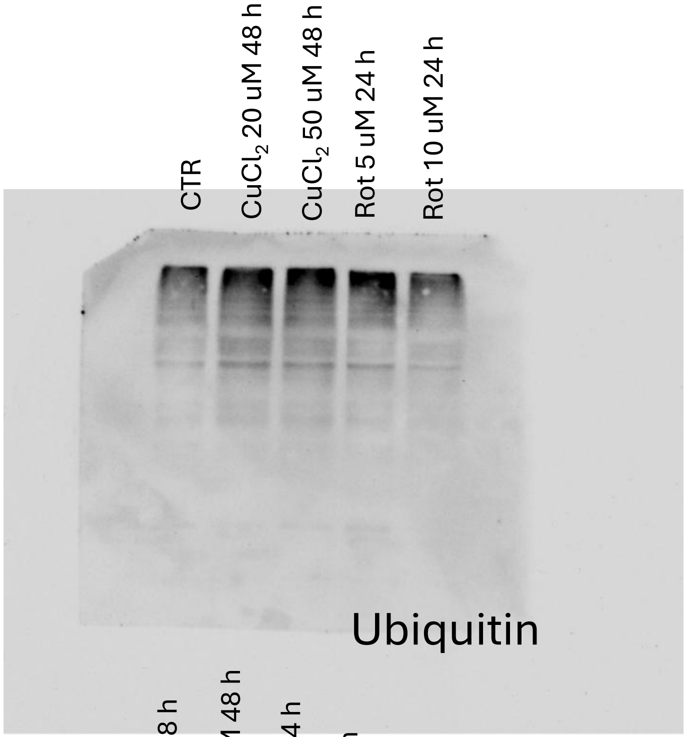

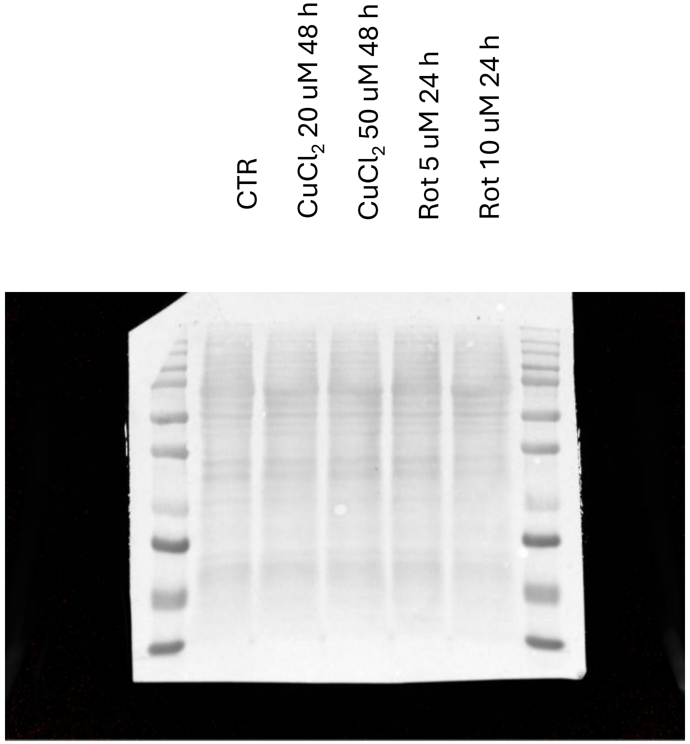

Ponceau S

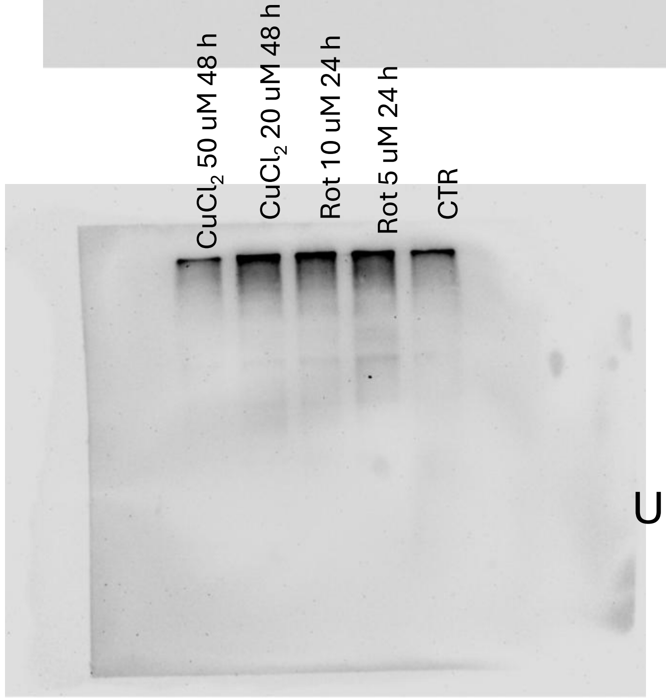

Ubiquitin

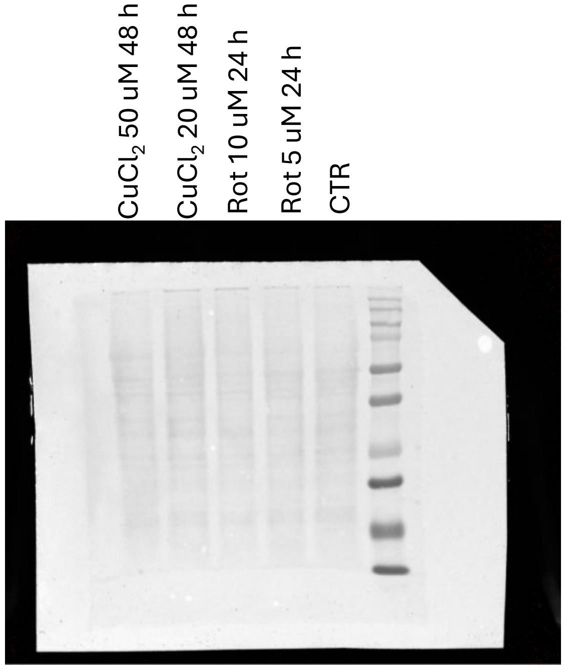

Ponceau S

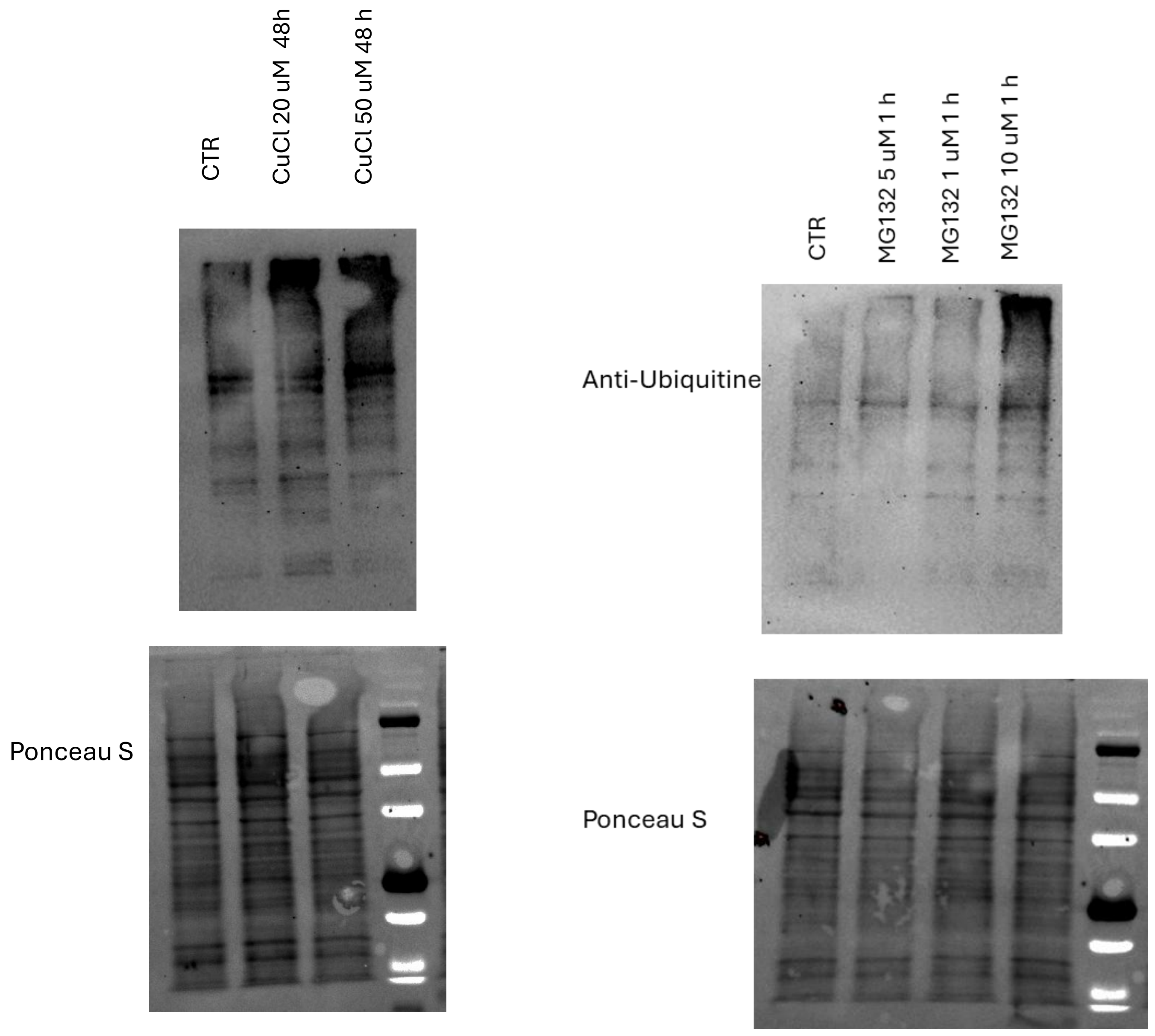
CTR
CuCl 20 uM 48h
CuCl 50 uM 48 h
Ponceau S
CTR
MG132 5 uM 1 h
MG132 1 uM 1 h
MG132 10 uM 1 h
Anti-Ubiquitine
Ponceau S

UBIQUITINE

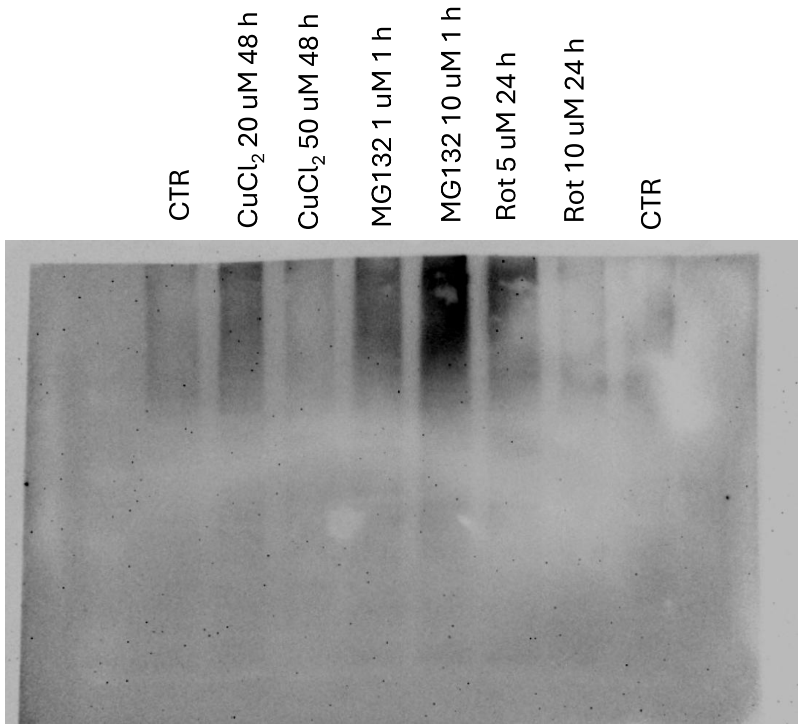

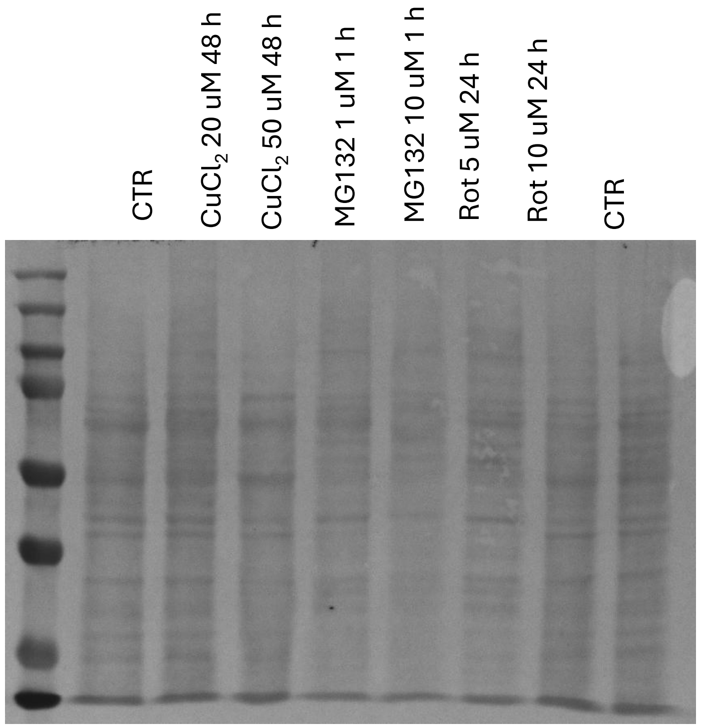

PONCEAU S

Raw data: P-S129-a-synuclein

Figure 7 A

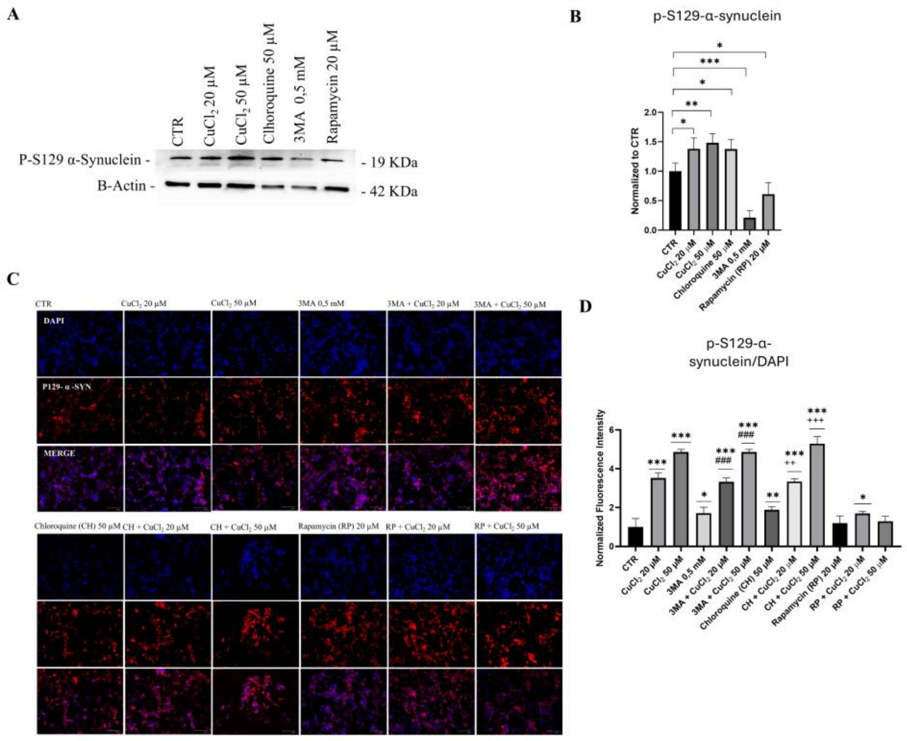

Figure 7. Effects of copper and autophagy modulators on p-S129-α-synuclein. Differentiated SH-SY5Y cells were treated with 20 or 50 µM CuCl2 for 48 h in combination with chloroquine (CH, 50 µM, 24 h), 3-MA (0.5 mM, 24 h), or rapamycin (RP, 20 µM, 24 h). (A) Representative immunoblots showing p-S129-α-synuclein protein levels. (B) Densitometric quantification of p-S129-α-synuclein normalized to β-actin (n = 4). (C) Representative fluorescence microscopy images of p-S129-α-synuclein (red). Merged images show colocalization of p-S129-α-synuclein with nuclei (blue). Magnification, 20×; scale bar, 125 µm. (D) Graphical representation of the fluorescence intensity of p-S129-α-synuclein normalized to nuclei (n = 3). Data are presented as mean ± SD. Statistical significance versus untreated control (CTR) was assessed by one-way ANOVA followed by Tukey's post-hoc test (α = 0.05). Analyses were performed using GraphPad Prism 8.0.1. * $p < 0.0332$; ** $p < 0.0021$; *** $p < 0.0002$. ++ $p< 0.0021$; +++ $p< 0.0002$ versus 3MA; ### $p < 0.0002$ versus CH

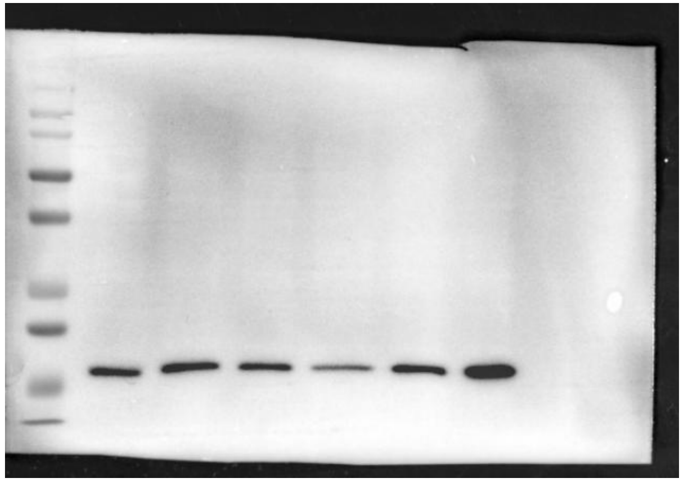

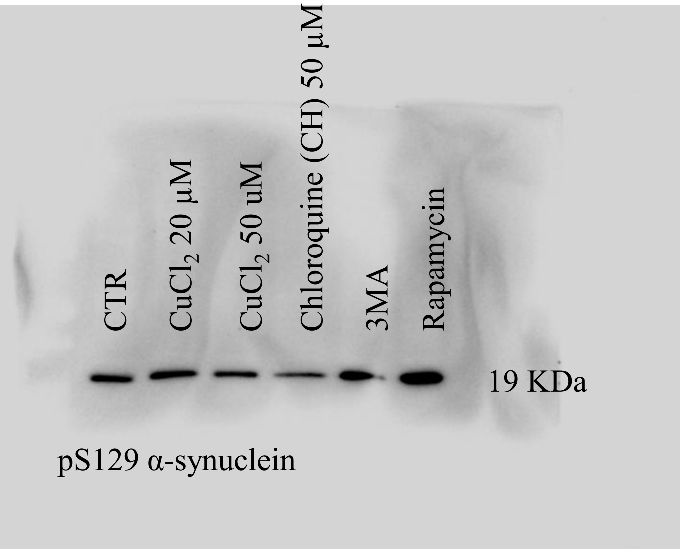
CTR
$CuCl_2$ 20 µM
$CuCl_2$ 50 uM
Chloroquine (CH) 50 µM
3MA
Rapamycin
19 KDa
pS129 α-synuclein

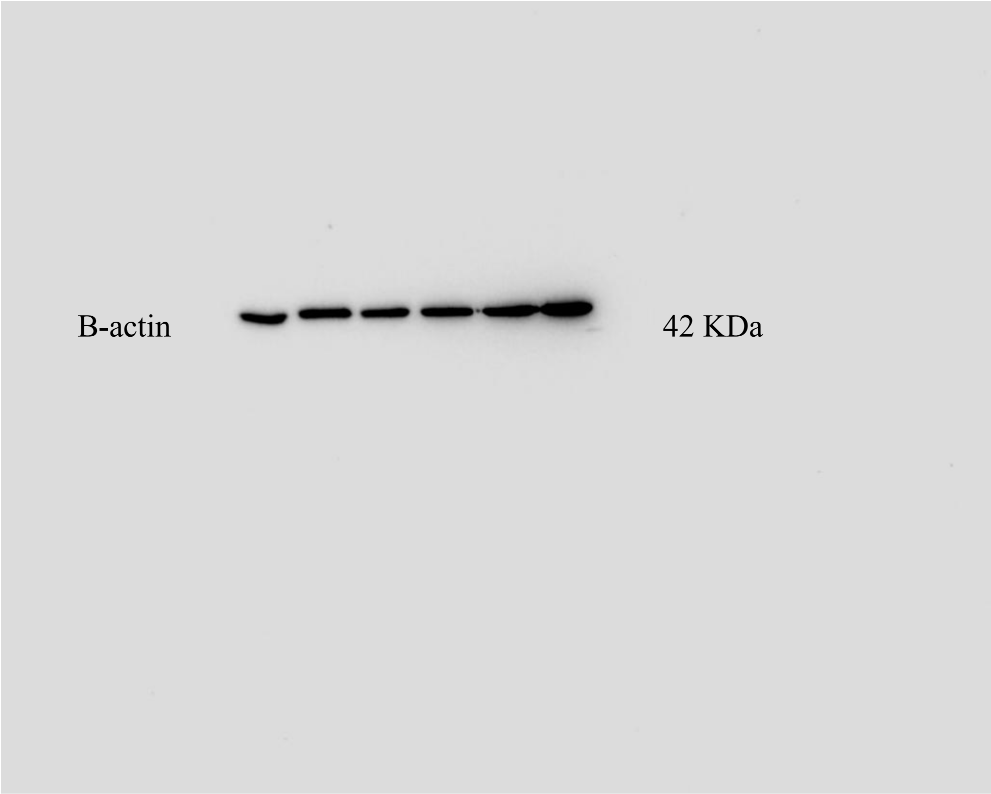
B-actin
42 KDa

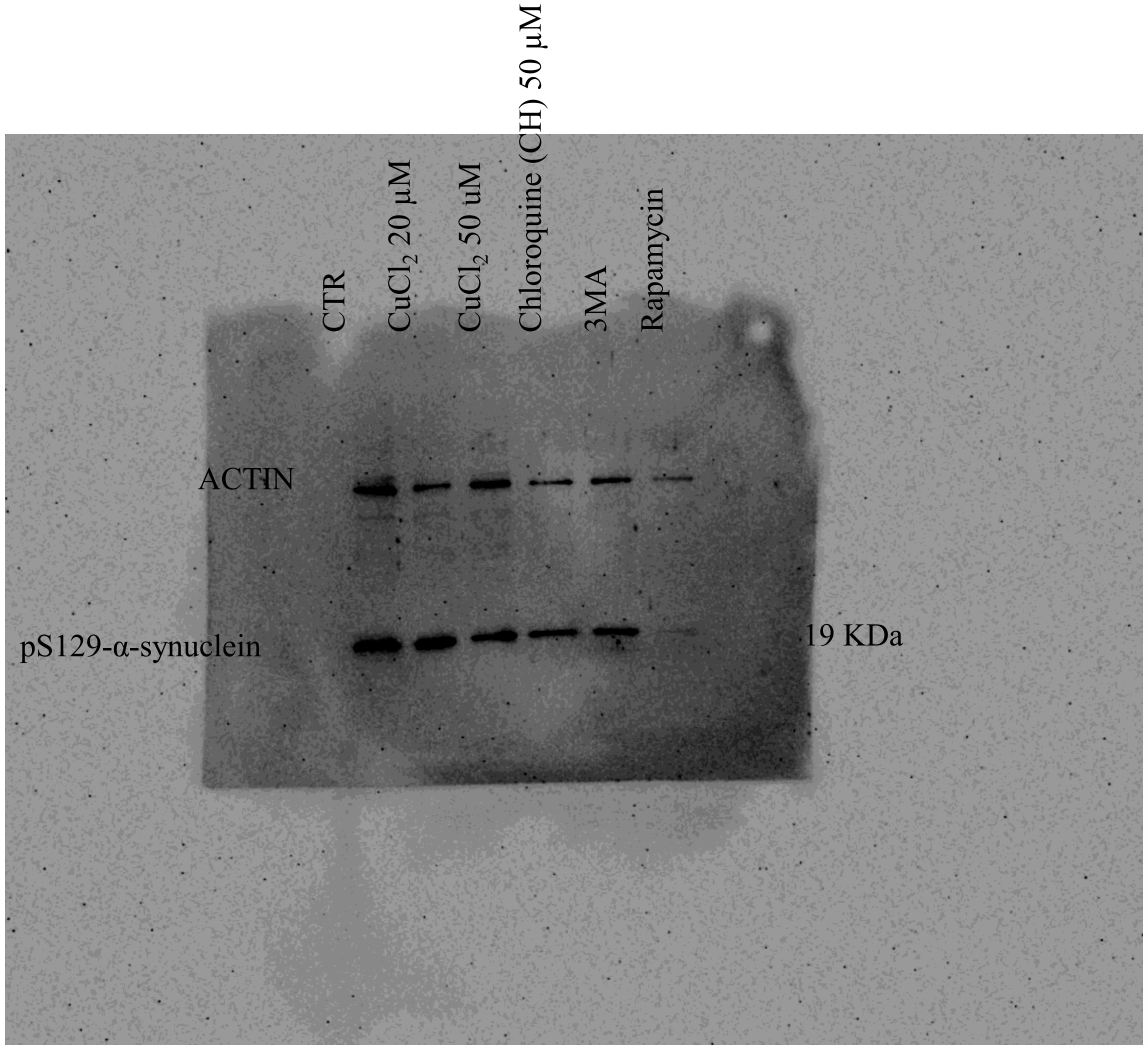
CTR
CuCl2 20 µM
CuCl2 50 uM
Chloroquine (CH) 50 µM
3MA
Rapamycin
ACTIN
pS129-α-synuclein
19 KDa

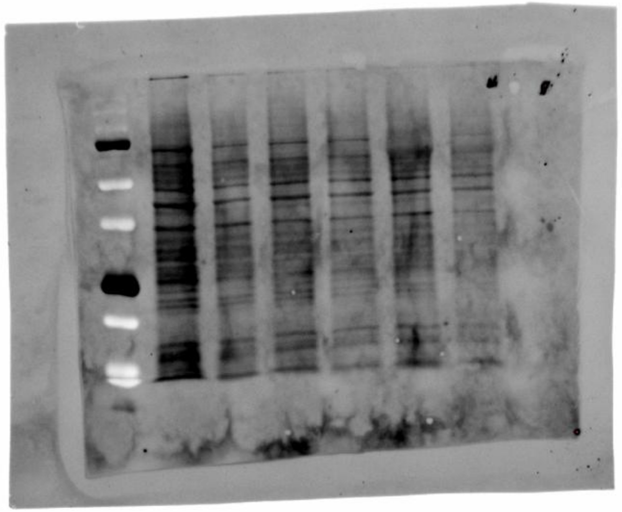

PONCEAU S

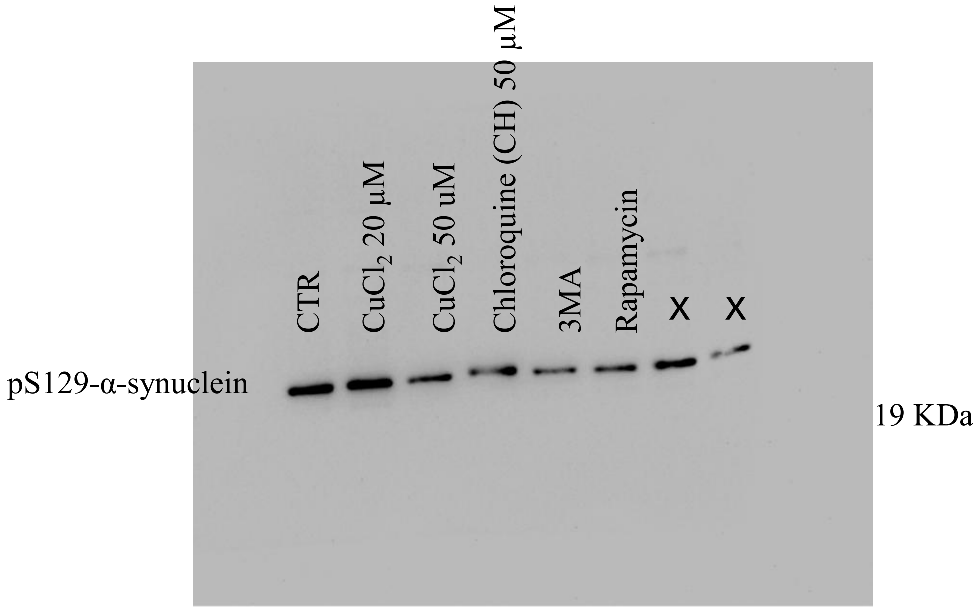
CTR
$CuCl_2$ 20 µM
$CuCl_2$ 50 uM
Chloroquine (CH) 50 µM
3MA
Rapamycin
X
X
pS129-α-synuclein
19 KDa

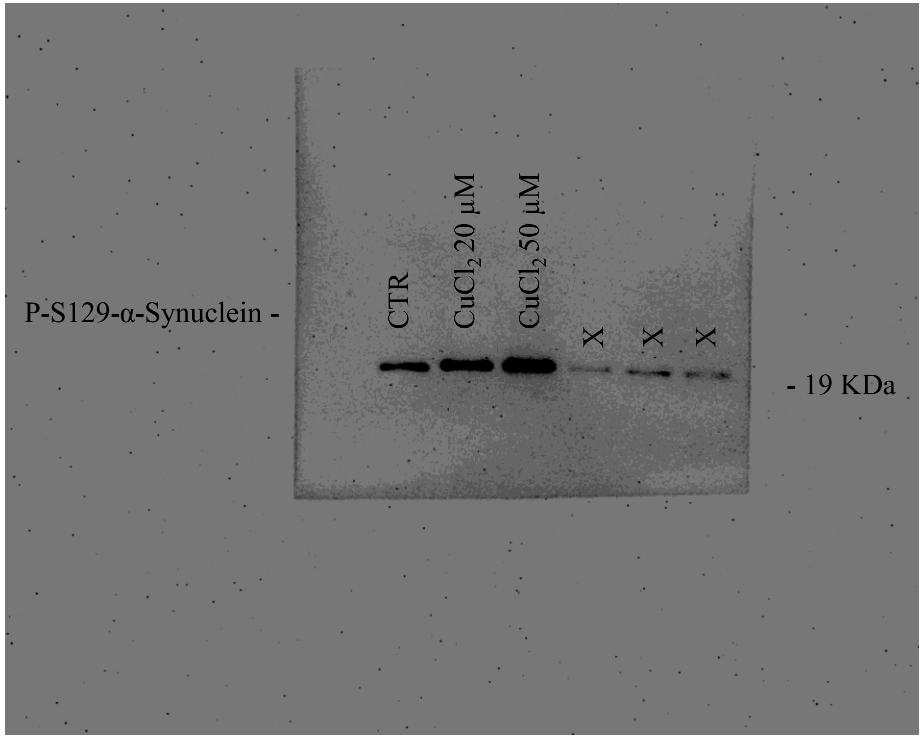
P-S129-α-Synuclein -
CTR
$CuCl_2$ 20 µM
$CuCl_2$ 50 µM
X
X
X
- 19 KDa

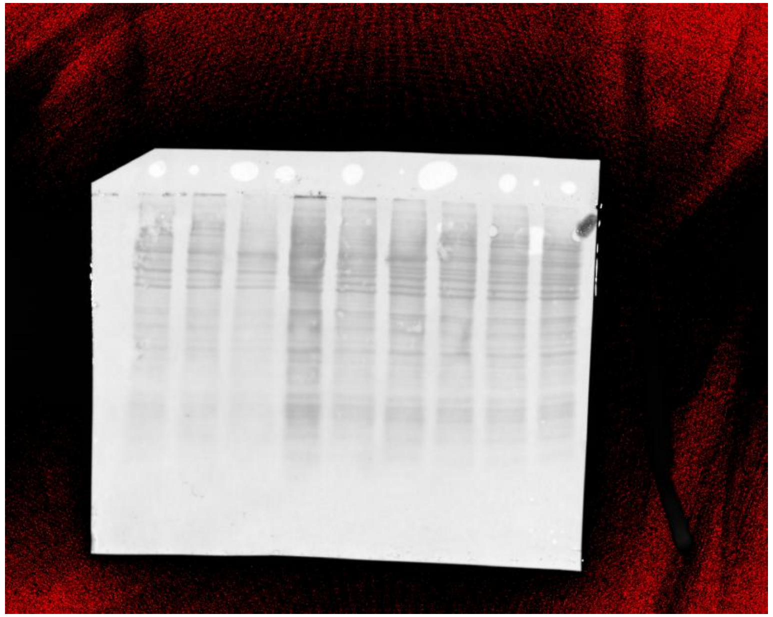

3

PONCEAU S

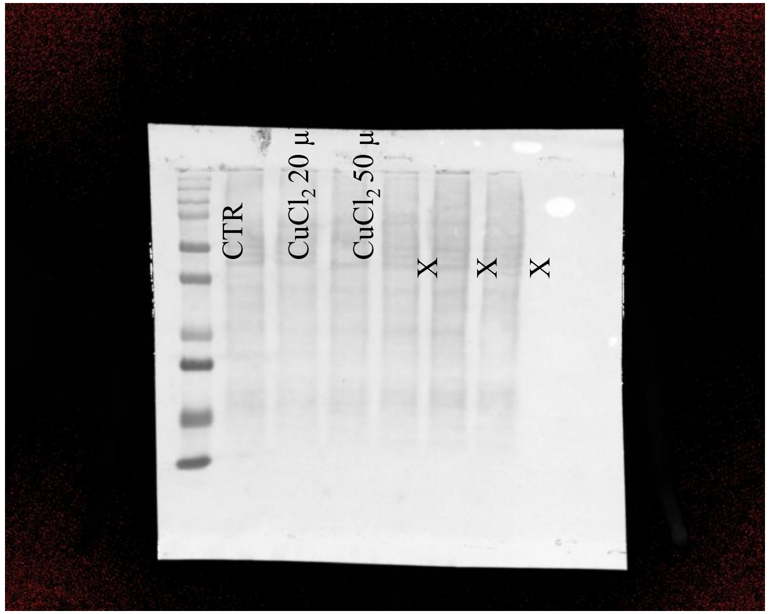
CTR
X
X
X

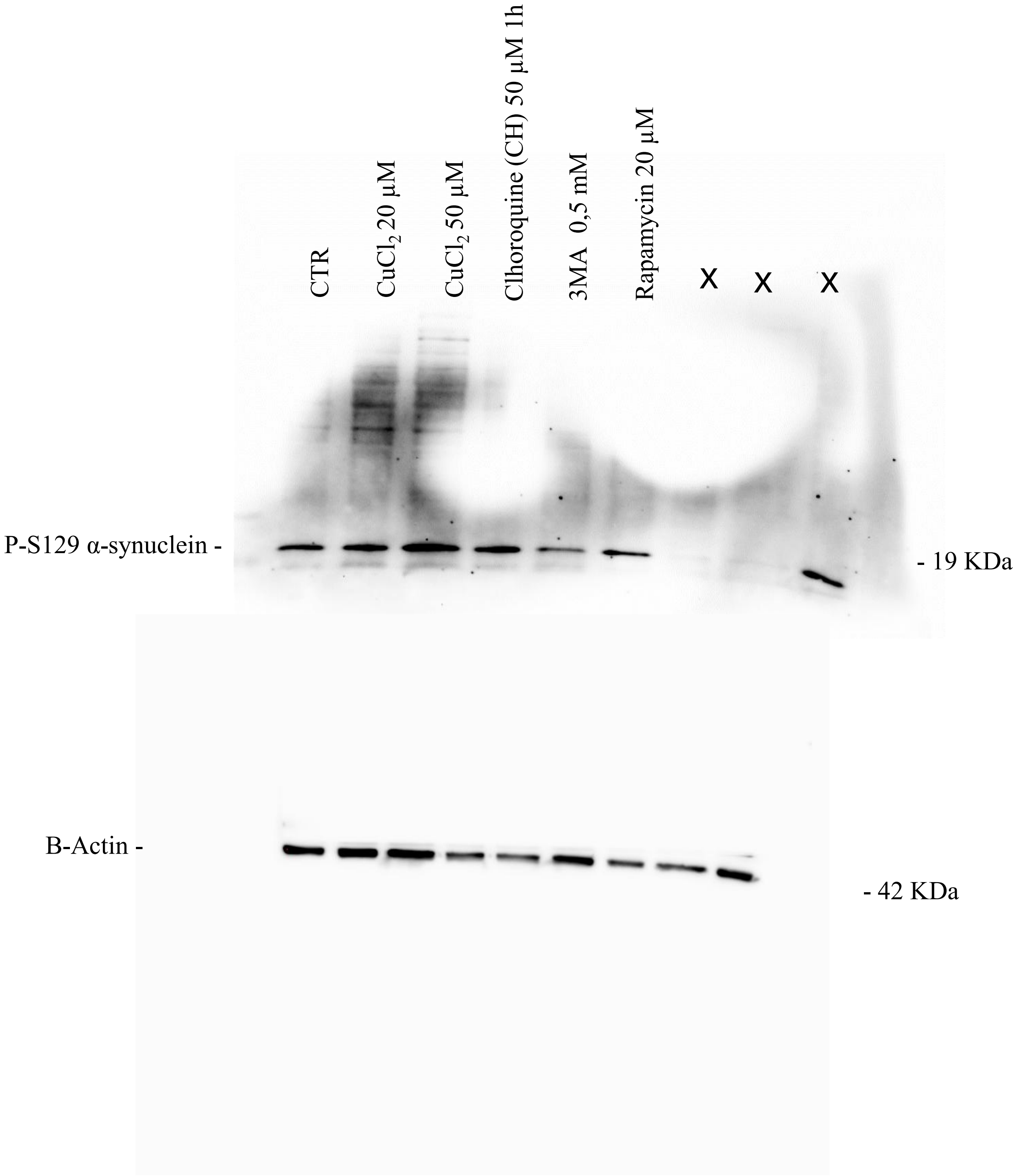
CTR
$CuCl_2$ 20 µM
$CuCl_2$ 50 µM
Clhoroquine (CH) 50 µM 1h
3MA 0,5 mM
Rapamycin 20 µM
X
X
X
P-S129 α-synuclein -
- 19 KDa
B-Actin -
- 42 KDa

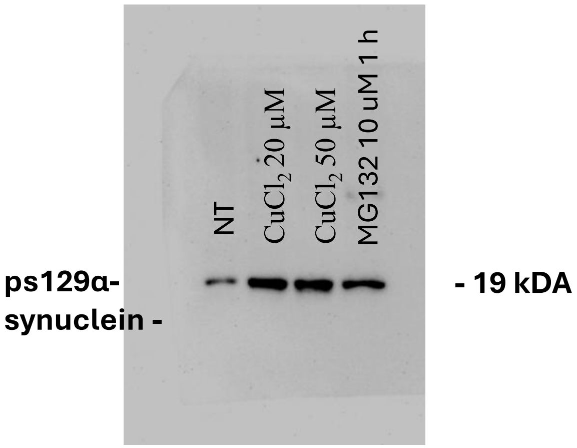
NT
$CuCl_2$ 20 µM
$CuCl_2$ 50 µM
MG132 10 uM 1 h
ps129α-synuclein -
- 19 kDA

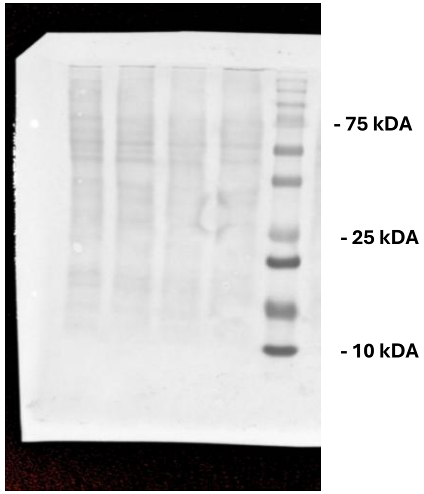
- 75 kDA
- 25 kDA
- 10 kDA
